# A systematic evaluation of double-expressor lymphoma: prognostic impact, determinants of outcome, and comparative efficacy and safety of novel therapies

**DOI:** 10.3389/fonc.2026.1823300

**Published:** 2026-05-21

**Authors:** Hao Guan, Zhihe Liu, Chengwen Gao, Wenqiu Wang, Kaiyue Liu, Xia Zhao

**Affiliations:** 1 Department of Lymphoma, Affiliated Hospital of Qingdao University, Qingdao, China; 2 Laboratory of Medical Biology, Medical Research Center, The Affiliated Hospital of Qingdao University, Qingdao, Shandong, China

**Keywords:** Bruton’s tyrosine kinase inhibitor, comparative effectiveness, diffuse large B-cell lymphoma, double-expressor lymphoma, network meta-analysis, prognostic factors, zanubrutinib

## Abstract

**Background:**

Double-expressor lymphoma (DEL), defined by concurrent MYC and BCL2 protein overexpression in diffuse large B-cell lymphoma (DLBCL), is associated with poor prognosis, but precise risk quantification and optimal treatments remain unclear.

**Methods:**

We conducted a two-stage evidence synthesis. First, a systematic review and meta-analysis quantified hazard ratios for progression-free survival (PFS) and overall survival (OS) comparing DEL with non-DEL and identified prognostic factors within DEL. Second, a network meta-analysis ranked the efficacy and safety of regimens including rituximab with cyclophosphamide, doxorubicin, vincristine, and prednisone (R-CHOP); dose-adjusted etoposide, prednisone, vincristine, cyclophosphamide, and doxorubicin plus rituximab (DA-EPOCH-R); R-CHOP plus lenalidomide (R2-CHOP); R-CHOP plus ibrutinib (I+R-CHOP); R-CHOP plus zanubrutinib (Z+R-CHOP); and R-CHOP plus venetoclax (Ven-R-CHOP).

**Results:**

In 66 studies (9,808 patients), DEL was significantly associated with inferior PFS (hazard ratio 1.78, 95% confidence interval 1.50–2.10) and OS (1.90, 1.68–2.15). Within DEL, high International Prognostic Index, advanced age, elevated lactate dehydrogenase, B symptoms, and advanced stage predicted inferior PFS and OS; TP53 mutation and poor Eastern Cooperative Oncology Group performance status predicted inferior OS. In 13 treatment studies (1,803 patients), Z+R-CHOP and Ven-R-CHOP showed the highest efficacy. Z+R-CHOP demonstrated favorable hematological toxicity compared with other novel regimens.

**Conclusion:**

DEL is a distinct high-risk subtype with quantifiable prognostic detriment. Z+R-CHOP emerges as a promising strategy requiring validation in prospective studies.

## 1 Introduction

Diffuse large B-cell lymphoma (DLBCL) is the most prevalent subtype of non-Hodgkin lymphoma (1). Epidemiological studies have identified diverse risk factors for DLBCL, encompassing genetic predisposition, clinical characteristics, immune dysregulation, and viral, environmental, or occupational exposure (2). The double-expressor phenotype is relatively frequent in DLBCL, observed in approximately 18% to 44% of cases (3). Double-expressor lymphoma (DEL), characterized by concurrent overexpression of MYC and BCL2 proteins, is recognized as a marker of poor prognosis in DLBCL, although it has not been classified as a distinct diagnostic entity. In the WHO Classification of Haematolymphoid Tumours, 5th edition (WHO-HAEM5) (2022) classification, DEL is retained within the “Diffuse large B-cell lymphoma, not otherwise specified (DLBCL, NOS)” category, defined by an immunohistochemical cutoff of ≥40% for MYC and >50% for BCL2. However, it is now specified as a distinct biologic subtype, underscoring its significance beyond a simple prognostic variable (4).

Of note, DEL must be rigorously distinguished from double-hit lymphoma (DHL), as these represent two biologically distinct entities (3). DEL is defined by concurrent MYC and BCL2 protein overexpression detected via immunohistochemistry, in the absence of corresponding gene rearrangements, rendering its diagnosis more straightforward than that of DHL. In contrast, DHL is characterized by concurrent MYC rearrangement with BCL2 and/or BCL6 rearrangements as confirmed by fluorescence *in situ* hybridization (FISH) and is classified in the WHO-HAEM5 classification as an independent diagnostic entity—”high-grade B-cell lymphoma with rearrangements of MYC, BCL2, and/or BCL6” (HGBL-DH/TH). DHL accounts for approximately 5% to 7% of DLBCL cases and is associated with an even poorer prognosis (3–5). Notably, although a subset of DHL cases may concurrently display the DEL phenotype, the majority of DEL cases lack the defining gene rearrangements of DHL (5).

Although the clinical significance of the double-expressor phenotype is well-recognized, notable gaps persist in the existing evidence. First, most studies have incorporated DEL merely as a covariate in multivariable prognostic models rather than conducting in-depth investigations specifically dedicated to this subtype. Consequently, the understanding of prognostic determinants operating uniquely within DEL remains insufficient. Second, driven by the absence of a recognized standard of care (6), current therapeutic research predominantly focuses on comparing intensified chemotherapy regimens [e.g., dose-adjusted etoposide, prednisone, vincristine, cyclophosphamide, and doxorubicin plus rituximab (DA-EPOCH-R)] or rituximab with cyclophosphamide, doxorubicin, vincristine, and prednisone (R-CHOP) combined with a single targeted agent (e.g., ibrutinib and lenalidomide) against standard R-CHOP. Direct head-to-head comparisons among these novel regimens are lacking. It is worth noting that emerging studies have indicated promising potential for next-generation Bruton’s tyrosine kinase (BTK) inhibitors (e.g., zanubrutinib (7)) and BCL2 inhibitors (e.g., venetoclax (8)). However, these data are fragmented, and there is a paucity of comparative evidence regarding both their relative efficacy and safety profiles.

This study first employed a meta-analysis to quantify the prognostic impact of DEL on DLBCL. Using progression-free survival (PFS) and overall survival (OS) as endpoints and hazard ratios (HRs) as the effect measure, we synthesized data from studies comparing DEL versus non-DEL outcomes. Simultaneously, we synthesized evidence on the prognostic value of various factors within the DEL population.

Subsequently, we identified all reports comparing treatment regimens for DEL patients. We conducted a network meta-analysis to rank the relative efficacy of these different therapeutic approaches. Finally, we summarized the adverse event profiles reported across these comparative treatment studies to evaluate the relative safety of the different regimens.

## 2 Methods

### 2.1 Selection criteria and search strategy

This systematic review and meta-analysis was conducted and reported in accordance with the Preferred Reporting Items for Systematic Reviews and Meta-Analyses (PRISMA) 2020 statement.

We systematically searched the PubMed, Embase, and Web of Science databases for relevant literature published between January 1, 2010, and December 20, 2025. The search strategy incorporated relevant keywords and Medical Subject Headings (MeSH) terms, including “Lymphoma, Large B-Cell, Diffuse”, “double expresser lymphoma”, and their variants (**Supplementary Table 1**).

An initial screening of titles and abstracts was performed. Studies were excluded if they were 1) case reports, 2) review articles, 3) focused solely on primary extranodal lymphomas, 4) mechanistic studies without clinical outcomes, 5) reports of transformation from other lymphoid malignancies, 6) duplicates, or 7) unpublished conference abstracts.

The inclusion criteria were defined using the PICOS framework.

1. Population (P): patients with newly diagnosed or relapsed/refractory DLBCL.
2. Study design (S): randomized controlled trials (RCTs) or prospective/retrospective cohort studies.
3. Intervention/exposure (I)

Prognostic meta-analysis: Double-expressor (DEL) status was primarily defined according to the WHO-HAEM5 classification as concurrent protein overexpression of MYC (≥40%) and BCL2 (>50%) by immunohistochemistry (IHC). For studies that applied different IHC thresholds or utilized alternative detection methods—such as gene expression profiling (e.g., cDNA-mediated Annealing, Selection, extension and Ligation (DASL)) for *MYC* and *BCL2* mRNA quantification, or composite scores like the H-score—the authors’ original definitions were followed to identify the DEL cohort. For analyses within the DEL subgroup, exposures included the following: TP53 mutation, DHL status, high-risk International Prognostic Index (IPI; >2), age > 60 years, female gender, Eastern Cooperative Oncology Group (ECOG) performance status >2, advanced Ann Arbor stage (III/IV), extranodal involvement, elevated lactate dehydrogenase (LDH) level, the presence of B symptoms, germinal center B-cell (GCB) subtype, and high Ki-67 expression (typically defined as ≥70%). A broad inclusion strategy was employed for all meta-analyses. Initially, all studies that classified patients into the DEL and non-DEL groups (for the comparative analysis) or reported on prognostic factors within a DEL cohort (for the factor analysis) based on their original criteria were included in the pooled analysis. Subsequently, to test the robustness of the findings and minimize heterogeneity introduced by definitional variance, sensitivity/subgroup analyses were performed restricted to studies that defined DEL using the standardized WHO-HAEM5 IHC criteria (MYC ≥ 40% and BCL2 > 50%).

Network meta-analysis: Treatment regimens included DA-EPOCH-R, R-CHOP plus lenalidomide (R2-CHOP), ibrutinib plus R-CHOP (I+R-CHOP), R-CHOP plus other Bruton’s tyrosine kinase inhibitors (R-CHOP+BKI), zanubrutinib plus R-CHOP (Z+R-CHOP), tucidinostat (chidamide) plus R-CHOP (CR-CHOP), and venetoclax plus R-CHOP (Ven-R-CHOP). Adverse event rates were also extracted.

4) Comparator (C)

Prognostic meta-analysis: Non-DEL status. Within DEL, comparators were as follows: TP53 wild type, non-DHL, low/intermediate-risk IPI (1–2), age ≤ 60 years, male gender, ECOG 0–1, early Ann Arbor stage (I/II), no extranodal involvement, normal LDH, the absence of B symptoms, non-GCB subtype, and Ki-67 expression below the defined high cutoff (e.g., <70%).

Network meta-analysis: standard R-CHOP regimen. The proportion of patients without adverse events was used for safety comparisons.

5) Outcome (O)

Prognostic meta-analysis: HRs with 95% confidence intervals (CIs) for PFS and OS.

Network meta-analysis: odds ratios (ORs) with 95% CIs for complete response (CR) rates.

### 2.2 Data analysis

#### 2.2.1 The first meta-analysis (prognostic impact)

The following descriptive data were extracted from each study: first author, publication year, country/region of the study population, study period, study design, treatment regimen(s) used, disease status (newly diagnosed or relapsed/refractory), total sample size, median age (with range), the percentage of female patients, and whether DHL cases were explicitly excluded. The methodological quality of observational studies was assessed using the modified Newcastle-Ottawa Scale (NOS).

For survival analysis, the following data were extracted: HRs with 95% CIs and p-values for PFS and OS comparing DEL vs. non-DEL.

Within the DEL subgroup, HRs with 95% CIs and p-values for PFS and OS were extracted for the following comparisons: high-risk vs. low/intermediate-risk IPI, TP53 mutation vs. wild-type, presence vs. absence of DHL, age >60 vs. ≤60 years, female vs. male, ECOG > 2 vs. 0–1, Ann Arbor stage III/IV vs. I/II, presence vs. absence of extranodal involvement, elevated vs. normal LDH, presence vs. absence of B symptoms, GCB vs. non-GCB subtype, and high vs. low Ki-67 expression.

Pooled hazard ratios were calculated using the RevMan software (version 5.4). Heterogeneity among studies was quantified using the I^2^ statistic. An I^2^ value ≤50% with a Cochran’s Q test p-value >0.1 was considered indicative of acceptable heterogeneity, and a fixed-effects model was used; otherwise, a random-effects model was applied. Publication bias was assessed visually using funnel plots and statistically using Egger’s test where appropriate. p-Value <0.05 was considered statistically significant for pooled effects.

#### 2.2.2 Network meta-analysis (treatment efficacy and safety)

For the network meta-analysis, the following data were extracted: first author, publication year, country, study period, design, specific treatment regimens and patient numbers in each arm, disease status, line of therapy, median age (range), the percentage of female patients, exclusion of DHL cases, and study quality (using NOS for observational studies).

The efficacy data extracted were the number of patients achieving a CR and the total number of evaluable patients in each treatment arm. For safety assessment, the number of patients experiencing any adverse event (or specific grade ≥3 events, if available) for each regimen was extracted.

The network meta-analysis was conducted using the Stata software (version 18.0) with appropriate packages. Both direct and indirect evidence were synthesized within a frequentist framework to generate ORs and their 95% CIs for CR rates, comparing all regimens. Treatment rankings were estimated using surface under the cumulative ranking curve (SUCRA) values. Safety outcomes were compared descriptively by synthesizing the reported adverse event rates across studies.

## 3 Results

### 3.1 The first meta-analysis

#### 3.1.1 Search and data extraction

**Figure 1:**
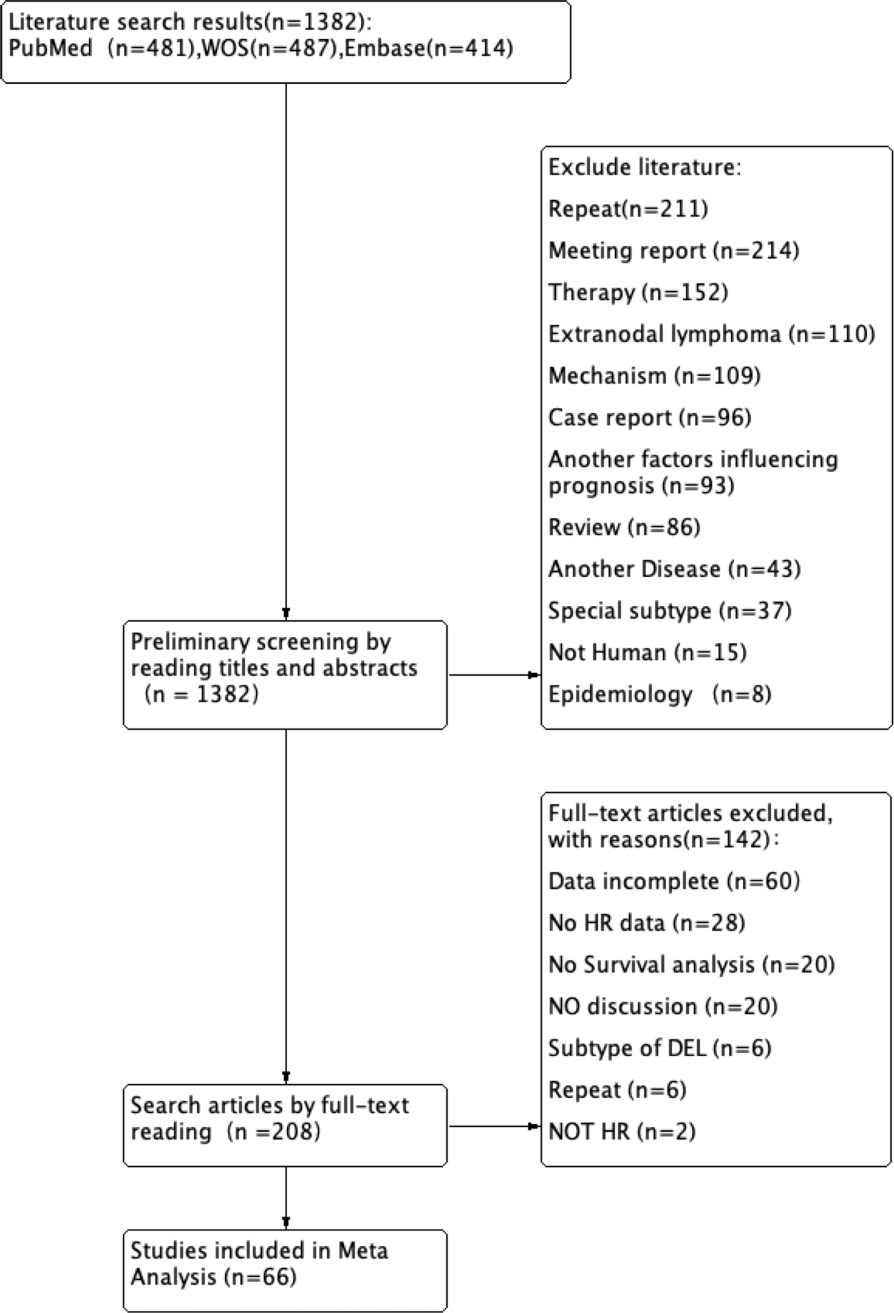
Study selection for the first meta-analysis. This PRISMA flow diagram illustrates the systematic process of literature identification, screening, eligibility assessment, and final inclusion of studies for the first pairwise meta-analysis. PRISMA, Preferred Reporting Items for Systematic Reviews and Meta-Analyses.

The initial database search yielded 1,382 records for screening. After removing 211 duplicate entries identified by DOI or by matching title, journal, authors, and publication date, 1,171 unique records underwent title and abstract screening. This resulted in 208 potentially eligible reports for full-text review. During the full-text assessment, studies were excluded based on the following criteria: 1) the absence of survival analysis, 2) primary survival endpoints other than PFS or OS, 3) effect measure not reported as an HR, 4) insufficient or incomplete data for extraction, 5) irrelevant study focus (e.g., not discussing the predefined exposures or outcomes), and 6) duplicates missed in the initial screening. This process culminated in the inclusion of 66 studies (**Figure 1**).

These 66 studies, encompassing 68 distinct patient cohorts with a total of 9,808 individuals, were included in the meta-analysis. The detailed characteristics of the included studies are summarized in **Table 1**.

**Table 1:** Characteristics of the studies included in the first meta-analysis.

Author	Country	Research type	Years recruiting	Therapy	N	Disease status	Median age (range)	Female	Exclude DHL?	NOS
Lili Wu (9)	China (CHN)	Cohort study	2016–2020	Chidamide+DICE	31	R/R	55 (27–71)	35.50%	Y	7
G. Vimalathas (10)	Denmark	Cohort study	2001–2018	R-CHOP or R-CHOP-like	111	NE	>60y, 63%	44%	N	8
Supanut Kumjan (11)	Thailand	Cohort study	2014–2021	R-CHOP/DA-EPOCH-R	177	NE	61 (21–93)	55.40%	Y	8
Jing Yuan Tan (12)	Singapore	Cohort study	2010–2022	R-CHOP/R-EPOCH	72	R/R	38.89%, >60y	44.44%	Y	7
XiaYin MS (13)	CHN	RCT	2020–2022	Z+R-CHOP	48	NE	59 (17–76)	39.60%	Y	
Yi-Fan Wu (14)	CHN	Cohort Jiangsu Province Hospital (JSPH)	2021–2023	R-CHOP-like	62	NE	>60y, 61.30%	53.20%	N	8
		Cohort (extra)	2021–2023	R-CHOP-like	284	NE	>60y, 63.20%	45.40%	N	8
Naree Warnnissorn (15)	Thailand	Cohort study	2014–2018	R-CHOP	111	NE	62 (26–88)	54%	N	8
Yohei Sasaki (16)	Japan	Cohort study	2008–2018	R-CHOP or R-CHOP-like	128	NE	71 (30–97)	49.20%	N	7
Jenny Coelho (17)	USA	Cohort study	2013–2019	R-CHOP	112	NE	45 (18–80)	38.20%	N	8
Tingting Yuan (18)	CHN	Cohort study	2017–2019	R-CHOP	289	NE	57 (18–83)	42.20%	N	7
Phuttirak Yimpak (19)	Thailand	Cohort study	2018–2019	R-CHOP	100	NE	62 (26–89)	52%	N	7
Xi Chen (20)	CHN	Cohort study	2016–2020	Chidamide+R-CHOP	62	NE	53.9 (19–77)	51.60%	Y	6
Yi-Zhuo Chen (21)	CHN	Cohort study	Unclear	Line 1 or line 2	130	NE	≥60y, 96.7%	49.23%	Y	7
Tan JY (22)	Singapore	Cohort study	2010–2022	High dose chemotherapy followed by ASCT (HDC+ASCT)	72	R/R	≥60y, 38.89%	44.44%	Y	7
Yanjie Wang (23)	CHN	Cohort study	2018–2021	R-CHOP or R-EPOCH	194	NE^1^	39.2%, ≥60y	40.10%	Y	7
Aleksei K. Koviazin (24)	Russian	Cohort study	2010–2019	R-CHOP/DA-EPOCH-R	52	NE^2^	53 (22–64)	54%	N	6
Sung H-J, (25)	S. Korea	Cohort study	2013–2021	R-CHOP	153	NE	64 (24–93)	52.90%	N	7
Taha Al-Juhaishi (26)	USA	Cohort study	2000–2018	Rituximab plus BEAM (Carmustine, Etoposide, Cytarabine, Melphalan) (R-BEAM)	267	R/R	60 (18.3–79.6)	35.58%	N	7
Juan Carlos (27)	USA	Cohort study	2010–2020	R-CHOP	177	NE	84.7%, >60y	48.60%	Y	5
Jing Zhan (28)	CHN	Cohort study	2015–2018	R-CHOP/DA-EPOCH-R	150	NE	44%, >60y	51.33%	N	7
Marta Rodríguez (29)	Spain	Cohort study	Unclear	R-CHOP or R-CHOP-like	197	NE	65 (17–91)	47.70%	N	8
Jin Roh (30)	S. Korea	Cohort study	2007–2016	R-CHOP	353	NE	58.5 (mean)	40.79%	Y	7
Jin Roh (31)	S. Korea	Cohort study	2006–2018	R-CHOP	269	NE	58 (48–68)	43.10%	Y	7
Annalisa Chiappella (32)	Italy	RCT	2006–2010	R-dose dense/HDC+ASCT	185	NE	49 (38–56)	46%	N	7
R.-O. Casasnovas (33)	19 countries	RCT	Unclear	Selinexor	79	R/R	>50% (>65y)	41.77%	Y	6
Anna Dodero (34)	Italy	Cohort study	2015–2020	DA-EPOCH-R	122	NE	59 (24–79)	38.50%	N	8
Shoichi Kimura (35)	Japan	Cohort study	1994–2019	Combined chemotherapy	111	NE	>60y, >50%	64.86%	N	7
Sugeshnee Pather (36)	South Africa	Cohort study	2013–2017	CHOP or R-CHOP	110	NE	44 (Interquartile Range (IQR): 16)	54%	Y	7
Xin Yang (37)	CHN	Cohort study	2011–2016	R-CHOP	163	NE	47.2%, >60y	45.40%	Y	9
Denisse Castro (38)	USA	Cohort study	2010–2015	R-(mini)-CHOP/R-EPOCH	73	NE	74% ≥60y	52%	N	7
S. Rungwittayatiwat (39)	Thailand	Cohort study	2015–2018	R-chemo	87	NE	72.4% ≥60y	58.70%	Y	7
Bingjie Fan (40)	CHN	Cohort study	2016–2020	R-CHOP	152	NE	60.5 (15−87)	46.05%	N	6
Dao-guang Chen (41)	CHN	Cohort study	2011–2018	CHOP or R-CHOP	56	NE^3^	>60y, 17.3%	37.80%	N	7
Mu-Chen Zhang (42)	CHN	Cohort study	2016–208	R-CHOP+tucidinostat	49	NE	67 (61–75)	41%	N	6
Jie Xu (43)	USA	Cohort study	2010–2016	R-CHOP	125	NE	62 (18–86)	35.20%	N	6
Zhiping Ma (44)	CHN	Cohort study	2015–2017	R-CHOP or R-CHOP-like	98	NE	55 (8–76)	42.86%	N	7
Yu Ri Kim (45)	S. Korea	Cohort study	2006–2018	R-CHOP	67	NE	52% (<65y)	40.30%	N	7
Bogyeong Han (46)	Korea	Cohort study	2013–2018	R-CHOP	388	NE	>50% (>60y)	41.49%	N	9
Maysaa Abdulla (47)	Sweden	Cohort study	2002–2016	R-CHOP or R-CHOP-like	249	NE	≥60y, 73%	40%	N	8
K.-C. Phang (48)	Canada	Cohort study	2004–2010	R-CHOP or R-CHOP-like	65	NE	60.16 (5–84)	44%	N	6
Xin-Yu Zhang (49)	CHN	Cohort study	2006–2015	DA-EPOCH-R/R-CHOP	398	NE	55 (14–84)	44.47%	N	7
Sixia Huang (50)	CHN	Cohort study	2014–2018	R-CHOP-E/R-EPOCH	130	NE	60 (2–86)	50.77%	N	6
Allison Barraclough (51)	Canada	Cohort study	2002–2013	R-CHOP-like	175	NE^4^	62 (19–89)	51%	N	7
Pu Huang (50)	CHN	Cohort study	2011–2016	CHOP/R-CHOP	174	NE	48.3% ≥60y	47.70%	Y	5
Ching Soon Teoh (52)	Malaysia	Cohort study	2012–2015	R-CHOP	71	NE	47.1% ≥60y	48.10%	N	7
K. T Prochazka (53)	Austria	Cohort study	Unclear	R-CHOP or R-CHOP-like	117	NE	68.4% >60y	56.41%	Y	8
Linyu Li (54)	CHN	Cohort study	2012–2015	R-CHOP-like	212	NE	58.5 (21–86)	46.20%	N	7
Alex F. Herrera (55)	USA	Cohort study	2000–2014	RIC/MAC	78	R/R	54 (24–69)	32%	N	6
Joshua Allen (56)	USA	Cohort study	2000–2015	R-ICE (111-ASCT)	167	R/R	60 (21–86)	39.52%	N	8
Ichiro Kawashima (57)	Japan	Cohort study	2000–2017	Hematopoietic stem Cell Transplantation (HCT)	60	R/R^5^	52 (20–68)	37%	N	7
Alex F. Herrera (58)	USA	Cohort study	2000–2013	ASCT	117	R/R	(30–76)	40%	Y	8
Wenjuan Yu (59)	CHN	Cohort study	2009–2015	CHOP or R-CHOP	223	NE	>60y, 32.4%	40.40%	Unclear	6
Annette M. Staiger (60)	German	Cohort study	2003–2005	R-CHOP	57	NE^6^	69 (61–79)	49%	Y	7
Wenli Yan (61)	CHN	Cohort study	2010–2014	CHOP or R-CHOP	148	NE	≥60y, 47.30%	43.92%	Unclear	6
H. Takahashi (62)	Japan	Cohort study	2001–2013	R-Double-CHOP	40	NE	53 (19–68)	45%	N	8
A.-S. Cottereau (63)	France	Cohort study	2004–2009	R-CHOP or R-CHOP-like	57	NE	66 (22–87)	46%	Unclear	7
K. M. C. Schneider (27)	USA	Cohort study	2002–2014	R-CHOP	69	NE	59%, >60y	51%	N	8
Ting-Xun Lu (64)	CHN	Cohort study	2006–2014	R-CHOP or R-CHOP-like	141	NE	41.8% ≥60y	34.80%	N	7
David W. Scott (65)	British	Cohort study	2003–2013	R-CHOP	330	NE	64 (16–92)	38%	N	7
Idun Fiskvik (66)	Norway	Cohort study	2004–2008	Rituximab plus CHOEP (Cyclophosphamide, Doxorubicin, Vincristine, Etoposide, Prednisone) (R-CHOEP)	67	NE	53 (18–64)	31%	N	8
Katsuhiro Miura (67)	Japan	Cohort study	2001–2013	Rituximab plus IVAD (Ifosfamide, Vincristine, Doxorubicin [Adriamycin], Dexamethasone) (R-IVAD)	38	R/R	64 (38–79)	39.50%	N	7
Anamarija M. Perry (68)	USA+CAN	Cohort study	Unclear	R-CHOP or R-CHOP-like	62	NE	53% ≥60y	45.00%	N	8
Carmen Bellas (69)	Spain	Cohort study	1996–2011	R-CHOP or R-CHOP-like	100	NE/R/R^7^	61 (18–88)	47.00%	N	7
Eun Ji Oh (70)	Korea	Cohort study	2005–2011	R-CHOP or R-CHOP-like	224	NE	>60y, 46.0%	39.30%	N	8
Shimin Hu (71)	29 centers	Cohort study	2005–2010	R-CHOP or R-CHOP-like	466	NE	>60y, 58.0%	42%	N	8
Nathalie A. Johnson (72)	5 countries	Cohort study	Unclear	R-CHOP (training)	167	NE	62 (17–92)	Unclear	N	7
		Cohort study		R-CHOP (validation)	140	NE	65 (19–90)	Unclear	N	7

N refers to the total sample size included in the report. Some studies did not report the median age and range, instead using the proportion of cases reporting being older or younger than a certain age as reported in the study. 1) All 194 patients had bulky disease. 2) All 52 patients had Ann Arbor stage IV. 3) All 185 patients underwent autologous stem cell transplantation (ASCT). 4) All 175 patients had Ann Arbor stage I or II. 5) This cohort was inferred to contain 60 patients based on the context. 6) This study compared all DEL cases against non-DEL cases of the activated B-cell (ABC) subtype only. 7) The 100 patients consisted of 85 NE and 15 R/R.

NE, newly diagnosed diffuse large B-cell lymphoma; R/R, recurrent/refractory diffuse large B-cell lymphoma; DHL, double-hit lymphoma; NOS, Newcastle-Ottawa Scale; RCT, randomized controlled trial; R-CHOP, rituximab with cyclophosphamide, doxorubicin, vincristine, and prednisone; DA-EPOCH-R, dose-adjusted etoposide, prednisone, vincristine, cyclophosphamide, and doxorubicin plus rituximab; Z+R-CHOP, zanubrutinib plus R-CHOP; DEL, double-expressor lymphoma.

Among the 66 included studies, 15 utilized immunohistochemistry thresholds that differed from the standard criteria of MYC (≥40%) and BCL2 (>50%). Of these, seven studies adopted a more stringent definition, requiring MYC ≥ 40% and BCL2 > 70% (10, 26, 47, 48, 53, 70, 71). The remaining eight studies employed varied thresholds or methods, specifically MYC/BCL2 = 40%/40% (29), 40%/30% (52), 50%/30% (68), 50%/50% (69), and 70%/50% (44) (one study each), as well as one study using the DASL assay (63) or the H-score method (66) as alternatives to conventional IHC cutoff values. Furthermore, six studies did not explicitly specify their IHC thresholds. For consistency, these were treated as having applied the widely accepted standard definition of MYC ≥ 40% and BCL2 > 50% in our analyses.

#### 3.1.2 DEL vs. non-DEL

A total of 40 cohorts from 39 studies reported PFS data for DEL versus non-DEL, encompassing 5,877 patients. Of these, 32 cohorts (5,242 patients) involved newly diagnosed DLBCL (11, 15–20, 23, 24, 28, 29, 37, 38, 42, 44–47, 49, 51, 53, 54, 60–66, 71, 72), while eight studies (635 patients) involved relapsed/refractory (R/R) DLBCL (9, 12, 22, 55–58, 67) (**Supplementary Table 2**). OS data for DEL versus non-DEL were reported in 54 cohorts (7,992 patients). This included 45 cohorts (7,278 patients) with newly diagnosed DLBCL (10, 11, 16–20, 23, 24, 28–32, 35–38, 41–52, 54, 59–66, 68–73) and nine studies (714 patients) with R/R DLBCL (9, 12, 22, 33, 55–58, 67) (**Supplementary Table 2**).

**Figure 2:**
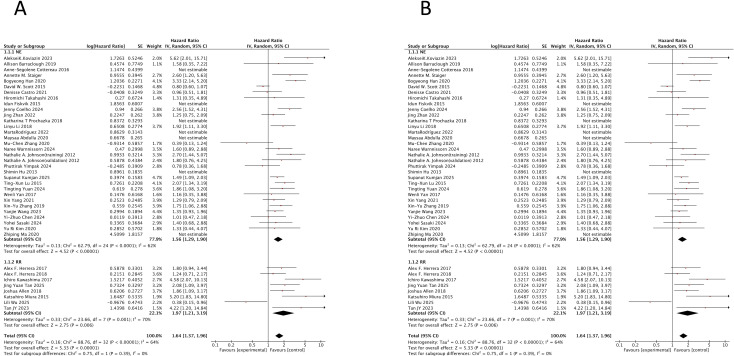
Prognostic impact of double-expressor lymphoma status on survival outcomes in diffuse large B-cell lymphoma. Forest plots comparing survival outcomes between double-expressor lymphoma (DEL) and non-double-expressor lymphoma (non-DEL) patients. **(A)** Progression-free survival (PFS). **(B)** Overall survival (OS). HR, hazard ratio; CI, confidence interval; DEL, double-expressor lymphoma; non-DEL, non-double-expressor lymphoma.

The meta-analysis demonstrated that DEL was associated with a significantly worse PFS compared to non-DEL, with a pooled HR of 1.78 (95% CI: 1.50–2.10; p < 0.00001). Substantial heterogeneity was observed among studies (I^2^ = 64%; p < 0.00001), although there was no significant heterogeneity between the newly diagnosed and R/R subgroups (p = 0.64). The adverse impact of DEL on PFS was consistent in both newly diagnosed DLBCL (HR = 1.74, 95% CI: 1.45–2.08; p < 0.00001; I^2^ = 64%) and R/R DLBCL (HR = 1.97, 95% CI: 1.21–3.19; p = 0.006; I^2^ = 70%). All pooled HRs were statistically significant (p < 0.05) (**Figure 2A**).

Similarly, DEL was associated with significantly inferior OS compared to non-DEL, with a pooled HR of 1.84 (95% CI: 1.69–2.00; p = 0.00002) and moderate heterogeneity (I^2^ = 45%; p = 0.00002). The negative prognostic impact on OS was confirmed in both newly diagnosed DLBCL (HR = 1.82, 95% CI: 1.67–1.99; p = 0.0001; I^2^ = 49%) and R/R DLBCL (HR = 1.94, 95% CI: 1.52–2.46; p = 0.28; I^2^ = 18%). No significant heterogeneity was found between these two clinical subgroups (p = 0.64). All results were statistically significant (**Figure 2B**).

Sensitivity analysis was performed to explore sources of heterogeneity. For the PFS analysis, the removal of specific studies (Bogyeong Han (46), David W. Scott (65), and Mu-Chen Zhang (R-CHOP+tucidinostat) (42) in the newly diagnosed group; Alex F. Herrera (RIC/MAC) (55) and Lili Wu (chidamide+DICE) (9) in the R/R group) led to a substantial reduction in heterogeneity (overall DEL, I^2^ = 38%, p = 0.01; newly diagnosed, I^2^ = 36%, p = 0.03; R/R: I^2^ = 31%, p = 0.20). For the OS analysis, removing studies by Aleksei K. Koviazin (24), Bogyeong Han (46), Idun Fiskvik (66), and Yi-Zhuo Chen (20) from the newly diagnosed group also reduced heterogeneity (overall DEL, I^2^ = 34%, p = 0.01; newly diagnosed, I^2^ = 37%, p = 0.009; R/R: I^2^ = 18%, p = 0.28).

Subsequently, we performed a sensitivity analysis by excluding studies that applied non-standard DEL definitions. In the first analysis, after excluding seven such studies (all involving newly diagnosed DLBCL patients), DEL remained a statistically significant predictor for inferior PFS in the overall population (HR = 1.64, 95% CI: 1.37–1.96, p < 0.00001; I^2^ = 64%) as well as in the newly diagnosed subgroup (HR = 1.56, 95% CI: 1.29–1.90, p < 0.0001; I^2^ = 62%). In a second, broader sensitivity analysis, exclusion of 12 studies with alternative definitions (also all in newly diagnosed DLBCL) yielded consistent results. DEL continued to show a significant association with worse PFS in the overall analysis (HR = 1.85, 95% CI: 1.68–2.03, p = 0.001; I^2^ = 44%) and in the newly diagnosed patient subgroup (HR = 1.83, 95% CI: 1.65–2.03, p = 0.00008; I^2^ = 49%).

#### 3.1.3 TP53 mutation (DEL-TP53+) vs. wild type (DEL-TP53−)

Five studies involving 247 patients with newly diagnosed DEL reported outcomes comparing TP53-mutated (TP53+) to TP53 wild-type (TP53−) disease (7, 14, 27, 34, 74) (**Supplementary Table 2**). Of note, TP53 alterations encompass both mutations and 17p deletions (del 17p). The present analysis was restricted to TP53 mutations alone. Although the adverse prognostic impact of 17p deletion in diffuse large B-cell lymphoma remains poorly defined, we were unable to identify eligible studies to formally evaluate this alteration (75).

**Figure 3:**
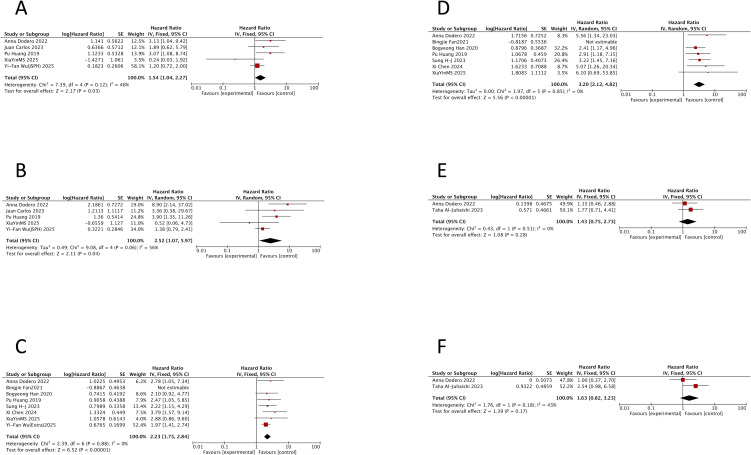
Prognostic impact of TP53 mutation, International Prognostic Index, and double-hit lymphoma status in double-expressor lymphoma. Forest plots showing hazard ratios for progression-free survival (PFS) and overall survival (OS) in patients with double-expressor lymphoma. **(A)** PFS for TP53 mutation versus wild type. **(B)** OS for TP53 mutation versus wild type. **(C)** PFS for high-risk (3–5) versus low/intermediate-risk (0–2) International Prognostic Index. **(D)** OS for high-risk versus low/intermediate-risk International Prognostic Index. **(E)** PFS for double-hit lymphoma-positive versus double-hit lymphoma-negative. **(F)** OS for double-hit lymphoma-positive versus double-hit lymphoma-negative. HR, hazard ratio; CI, confidence interval; IPI, International Prognostic Index; DHL, double-hit lymphoma.

The meta-analysis showed that TP53 mutation was associated with a significantly worse OS (HR = 2.52, 95% CI: 1.07–5.97; p = 0.04), albeit with notable heterogeneity (I^2^ = 56%; p = 0.06). After excluding the study by Yi-Fan Wu (Jiangsu Province Hospital) (14), heterogeneity was reduced (I^2^ = 33%; p = 0.21), and the association with OS became stronger (HR = 3.53, 95% CI: 1.33–9.39; p = 0.01) (**Figure 3A**). For PFS, TP53 mutation also demonstrated a significant adverse impact in the pooled analysis (HR = 1.54, 95% CI: 1.04–2.27; p = 0.03) with acceptable heterogeneity (I^2^ = 46%; p = 0.12) (**Figure 3B**).

Evidently, TP53 mutation confers a consistently adverse prognostic impact on both PFS and OS. Interestingly, however, the study by Xia Yin MS (7) reported HRs below 1 for both endpoints, representing a notable exception to this overall trend. This study was the only investigation to incorporate a novel agent, employing a Z+R-CHOP regimen. These findings provide valuable preliminary evidence suggesting that zanubrutinib may mitigate the poor prognostic impact of TP53 mutation in DEL.

#### 3.1.4 High IPI (DEL-IPI 3–5) vs. low IPI (DEL-IPI 0–2)

Eight studies involving 801 patients with newly diagnosed DEL compared outcomes between the high (3–5) and low/intermediate (0–2) IPI risk groups (7, 14, 21, 25, 34, 40, 46, 74) (**Supplementary Table 2**).

A high IPI score was associated with significantly inferior PFS (HR = 2.01, 95% CI: 1.36–2.97; p = 0.0005) (**Figure 3C**) and OS (HR = 2.58, 95% CI: 1.41–4.75; p = 0.002) (**Figure 3D**). Substantial heterogeneity was observed for both endpoints (PFS, I^2^ = 53%, p = 0.04; OS, I^2^ = 54%, p = 0.04).

Sensitivity analysis identified the study by Bingjie Fan (40) as a major source of heterogeneity. Its exclusion eliminated heterogeneity (PFS, I^2^ = 0%, p = 0.88; OS, I^2^ = 0%, p = 0.85) and strengthened the associations (PFS HR = 2.23, 95% CI: 1.75–2.84, p < 0.00001; OS HR = 3.20, 95% CI: 2.12–4.82, p < 0.0001). It is important to note an inherent limitation: the IPI risk group definitions varied slightly across the included studies (e.g., 3–5 vs. 0–2, 3–4 vs. 0–2, 4–5 vs. 0–1).

#### 3.1.5 DEL with DHL (DEL-DHL+) vs. DEL without DHL (DEL-DHL−)

Only two studies, involving a total of 291 DEL patients, compared outcomes between those with and without concurrent DHL (26, 34) (**Supplementary Table 2**). One study involved newly diagnosed patients but applied non-standard diagnostic criteria for DHL (34), whereas the other involved R/R patients using conventional criteria (26).

The pooled analysis showed that the presence of DHL within DEL was not significantly associated with PFS (HR = 1.43, 95% CI: 0.75–2.73; p = 0.28) (**Figure 3E**) or OS (HR = 1.63, 95% CI: 0.82–3.23; p = 0.17) (**Figure 3F**). This lack of significance is likely due to the very limited number of studies.

#### 3.1.6 Age > 60 years vs. age ≤ 60 years within DEL

**Figure 4:**
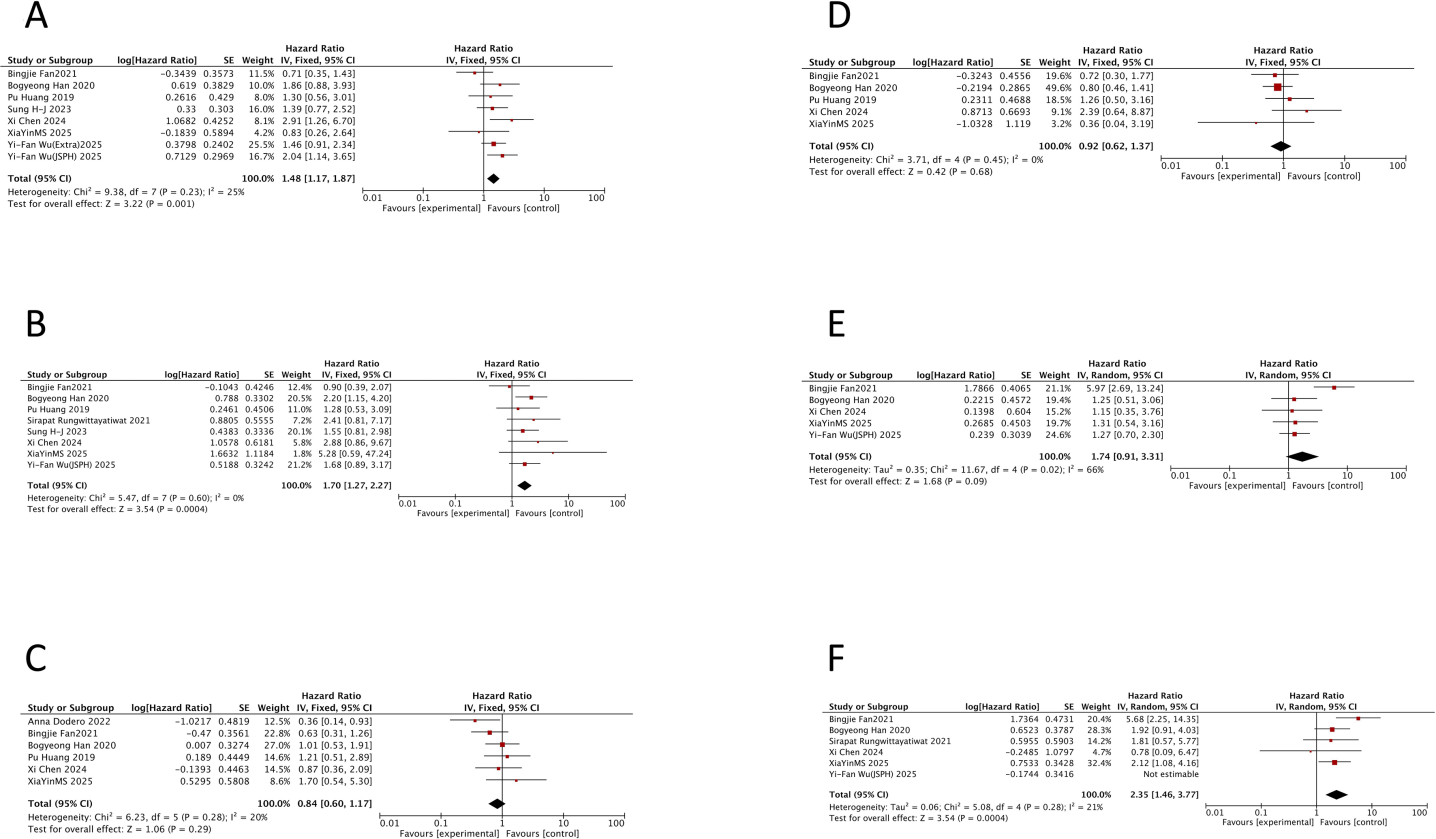
Prognostic impact of age, gender, and Eastern Cooperative Oncology Group performance status in double-expressor lymphoma. Forest plots showing hazard ratios for progression-free survival (PFS) and overall survival (OS) in patients with double-expressor lymphoma. **(A)** PFS for age >60 versus ≤60 years. **(B)** OS for age >60 versus ≤60 years. **(C)** PFS for female versus male. **(D)** OS for female versus male. **(E)** PFS for Eastern Cooperative Oncology Group performance status >2 versus 0–1/2. **(F)** OS for Eastern Cooperative Oncology Group performance status >2 versus 0–1/2. HR, hazard ratio; CI, confidence interval; ECOG, Eastern Cooperative Oncology Group.

PFS data comparing patients >60 versus ≤60 years within DEL were available from eight cohorts across seven studies (907 patients), all involving newly diagnosed disease (7, 14, 21, 25, 40, 46, 74) (**Supplementary Table 2**). Meta-analysis showed that age > 60 years was associated with significantly worse PFS (HR = 1.48, 95% CI: 1.17–1.87; p = 0.001) (**Figure 4A**). OS data were available from eight studies (717 patients) (7, 14, 21, 25, 39, 40, 46, 74) for the same comparison in newly diagnosed DEL (**Supplementary Table 2**). The analysis confirmed that older age was also a significant adverse prognostic factor for OS (HR = 1.70, 95% CI: 1.27–2.27; p = 0.0004) (**Figure 4B**). No significant heterogeneity was observed for either endpoint (PFS, I^2^ = 25%, p = 0.23; OS, I^2^ = 0%, p = 0.60).

#### 3.1.7 Female vs. male within DEL

Six studies involving 666 patients with newly diagnosed DEL compared PFS between female and male patients (7, 21, 34, 40, 46, 74) (**Supplementary Table 2**). The pooled analysis found no significant difference in PFS between female and male patients (HR = 0.84, 95% CI: 0.60–1.17; p = 0.29) (**Figure 4C**). Similarly, analysis of OS data from five studies (544 patients) (7, 21, 40, 46, 74) showed no significant association with gender (HR = 0.92, 95% CI: 0.62–1.37; p = 0.68) (**Figure 4D**). The lack of significance may be because gender was not a primary focus in any of the included studies.

#### 3.1.8 ECOG performance status >2 vs. 0–1/2 within DEL

PFS data comparing ECOG performance status >2 vs. 0–1/2 within DEL were available from five studies (430 newly diagnosed patients) (7, 14, 21, 40, 46) (**Supplementary Table 2**). The analysis showed a trend toward worse PFS for patients with ECOG > 2, but it did not reach statistical significance (HR = 1.74, 95% CI: 0.91–3.31; p = 0.09) (**Figure 4E**). OS data for the same comparison were available from six studies (517 newly diagnosed patients) (7, 14, 21, 39, 40, 46) (**Supplementary Table 2**). For OS, an ECOG status > 2 was associated with a significantly increased risk (HR = 1.86, 95% CI: 1.06–3.27; p = 0.03) (**Figure 4F**), although with substantial heterogeneity (I^2^ = 57%; p = 0.04). Sensitivity analysis indicated that removing the study by Yi-Fan Wu (JSPH) (33) reduced heterogeneity (I^2^ = 21%; p = 0.28).

#### 3.1.9 Advanced Ann Arbor stage (III/IV) vs. early stage (I/II) within DEL

**Figure 5:**
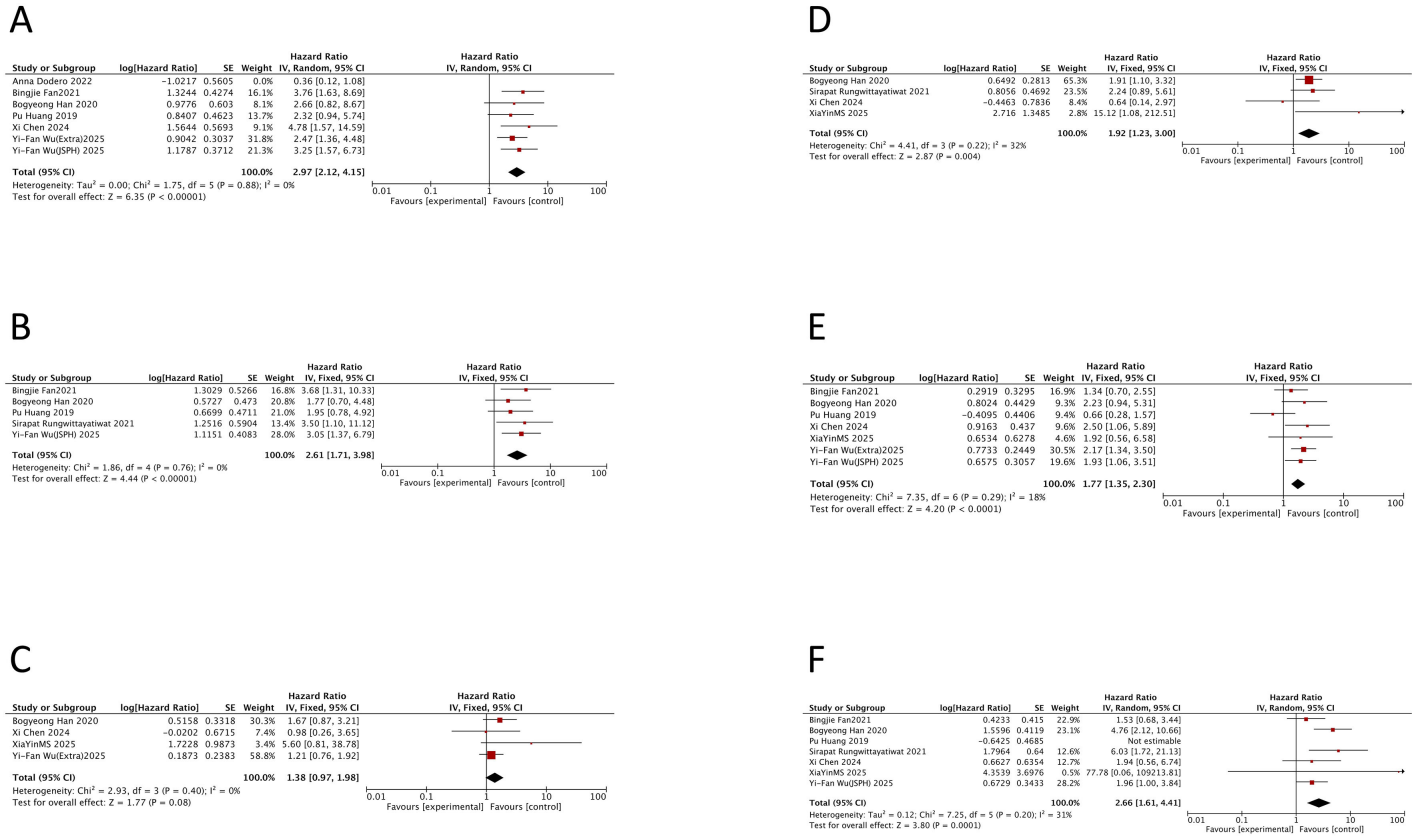
Prognostic impact of Ann Arbor stage, extranodal involvement, and lactate dehydrogenase level in double-expressor lymphoma. Forest plots showing hazard ratios for progression-free survival (PFS) and overall survival (OS) in patients with double-expressor lymphoma. **(A)** PFS for Ann Arbor stage III/IV versus I/II. **(B)** OS for Ann Arbor stage III/IV versus I/II. **(C)** PFS for presence versus absence of extranodal involvement. **(D)** OS for presence versus absence of extranodal involvement. **(E)** PFS for elevated versus normal lactate dehydrogenase. **(F)** OS for elevated versus normal lactate dehydrogenase. HR, hazard ratio; CI, confidence interval; LDH, lactate dehydrogenase.

PFS data comparing advanced (III/IV) versus early (I/II) Ann Arbor stage within DEL were available from seven cohorts across six studies (955 newly diagnosed patients) (14, 21, 34, 40, 46, 74) (**Supplementary Table 2**). The advanced stage (III/IV) was associated with significantly worse PFS compared to the early stage (I/II) (HR = 2.37, 95% CI: 1.40–3.99; p = 0.0001) (**Figure 5A**). Sensitivity analysis showed that removing the study by Anna Dodero (34) eliminated heterogeneity (I^2^ = 0%; p = 0.88). OS data for this comparison were reported in five studies (581 newly diagnosed patients) (14, 39, 40, 46, 74) (**Supplementary Table 2**). Similarly, the advanced stage was a strong predictor of inferior OS (HR = 2.61, 95% CI: 1.71–3.98; p < 0.0001) (**Figure 5B**) with no observed heterogeneity (I^2^ = 0%; p = 0.76).

#### 3.1.10 DEL with extranodal involvement vs. without extranodal involvement

Four studies (502 patients) reported PFS (7, 14, 21, 46), and four studies (305 patients) reported OS, comparing DEL patients with versus without extranodal involvement (7, 21, 39, 46). All patients had a newly diagnosed disease (**Supplementary Table 2**). Pooled analysis indicated that extranodal involvement was not significantly associated with PFS (HR = 1.38, 95% CI: 0.97–1.98; p = 0.08) (**Figure 5C**). In contrast, it was significantly associated with inferior OS (HR = 1.92, 95% CI: 1.23–3.00; p = 0.004) (**Figure 5D**). Notably, the definition of extranodal involvement differed across studies: two studies (Xia Yin MS (7) and Bogyeong Han (46)) defined it as involvement of ≥2 extranodal sites, while the others defined it as any extranodal involvement.

Beyond the quantitative extent of extranodal involvement, the specific anatomical site of disease dissemination merits careful consideration. Central nervous system involvement, for example, undoubtedly exerts a substantial adverse impact on prognosis (76). Regrettably, however, the available literature identified in our review was limited to studies examining the prognostic significance of extranodal site number, with no eligible studies evaluating the effect of specific involvement sites.

#### 3.1.11 Elevated LDH vs. normal LDH within DEL

PFS data comparing elevated versus normal LDH levels within DEL were available from seven cohorts across six studies (884 newly diagnosed patients) (7, 14, 21, 40, 46, 74). Elevated LDH was associated with significantly worse PFS (HR = 1.77, 95% CI: 1.35–2.30; p < 0.0001) with low heterogeneity (I^2^ = 18%; p = 0.29) (**Figure 5E**). OS data were available from seven studies (687 newly diagnosed patients) (7, 14, 21, 39, 40, 46, 74) (**Supplementary Table 2**). Elevated LDH was also a significant prognostic factor for worse OS (HR = 2.10, 95% CI: 1.09–4.07; p = 0.03), although with substantial heterogeneity (I^2^ = 65%; p = 0.03) (**Figure 5F**). Exclusion of the study by Pu Huang (74) reduced heterogeneity (I^2^ = 31%; p = 0.20).

#### 3.1.12 Presence vs. absence of B symptoms within DEL

**Figure 6:**
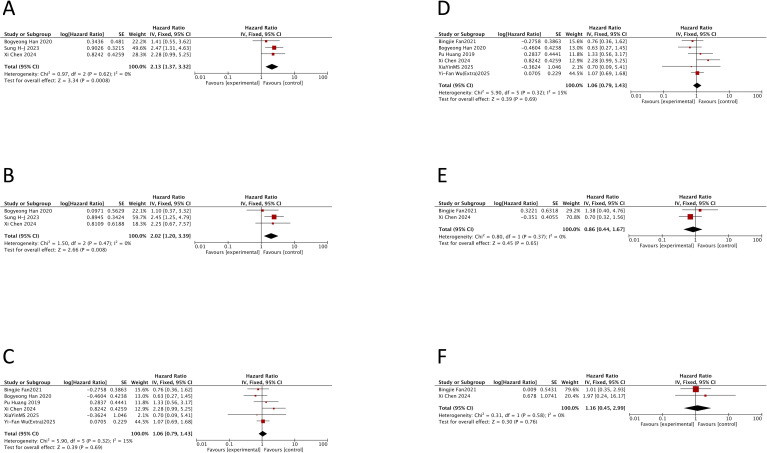
Prognostic impact of B symptoms, cell of origin, and Ki-67 proliferation index in double-expressor lymphoma. Forest plots showing hazard ratios for progression-free survival (PFS) and overall survival (OS) in patients with double-expressor lymphoma. **(A)** PFS for presence versus absence of B symptoms. **(B)** OS for presence versus absence of B symptoms. **(C)** PFS for germinal center B-cell-like versus non-germinal center B-cell-like subtype. **(D)** OS for germinal center B-cell-like versus non-germinal center B-cell-like subtype. **(E)** PFS for high versus low Ki-67 expression. **(F)** OS for high versus low Ki-67 expression. HR, hazard ratio; CI, confidence interval; GCB, germinal center B-cell; non-GCB, non-germinal center B-cell.

Three studies involving 323 patients with newly diagnosed DEL reported outcomes based on the presence or absence of B symptoms (21, 25, 46) (**Supplementary Table 2**). The presence of B symptoms was associated with significantly worse PFS (HR = 2.13, 95% CI: 1.37–3.32; p = 0.0008) (**Figure 6A**) and OS (HR = 2.02, 95% CI: 1.20–3.39; p = 0.008) (**Figure 6B**). No heterogeneity was observed for either endpoint (PFS, I^2^ = 0%, p = 0.62; OS, I^2^ = 0%, p = 0.47).

#### 3.1.13 DEL-GCB vs. non-GCB

Cell of origin (GCB vs. non-GCB) was evaluated in six studies for PFS (822 patients) (7, 14, 21, 40, 46, 74) and six studies for OS (625 patients) (7, 21, 39, 40, 46, 74), all in newly diagnosed DEL (**Supplementary Table 2**). The GCB subtype, compared to the non-GCB subtype, was not significantly associated with PFS (HR = 1.06, 95% CI: 0.79–1.43; p = 0.69) (**Figure 6C**) or OS (HR = 0.90, 95% CI: 0.60–1.37; p = 0.64) (**Figure 6D**) within the DEL population.

#### 3.1.14 High Ki-67 vs. low Ki-67 expression within DEL

Only two studies involving 214 patients with newly diagnosed DEL reported outcomes based on high versus low Ki-67 expression (21, 40) (**Supplementary Table 2**). High Ki-67 expression was not significantly associated with PFS (HR = 0.86, 95% CI: 0.44–1.67; p = 0.65) (**Figure 6E**) or OS (HR = 1.16, 95% CI: 0.45–2.99; p = 0.76) (**Figure 6F**). Furthermore, the cutoff values defining “high” Ki-67 differed between the two studies (>70% (40) vs. >80% (21)).

#### 3.1.15 Publication bias

Funnel plot analysis for each meta-analysis did not reveal significant publication bias for the comparisons that yielded statistically significant results (**Supplementary Figure 1**).

### 3.2 Network meta-analysis

#### 3.2.1 Search and data extraction

**Figure 7:**
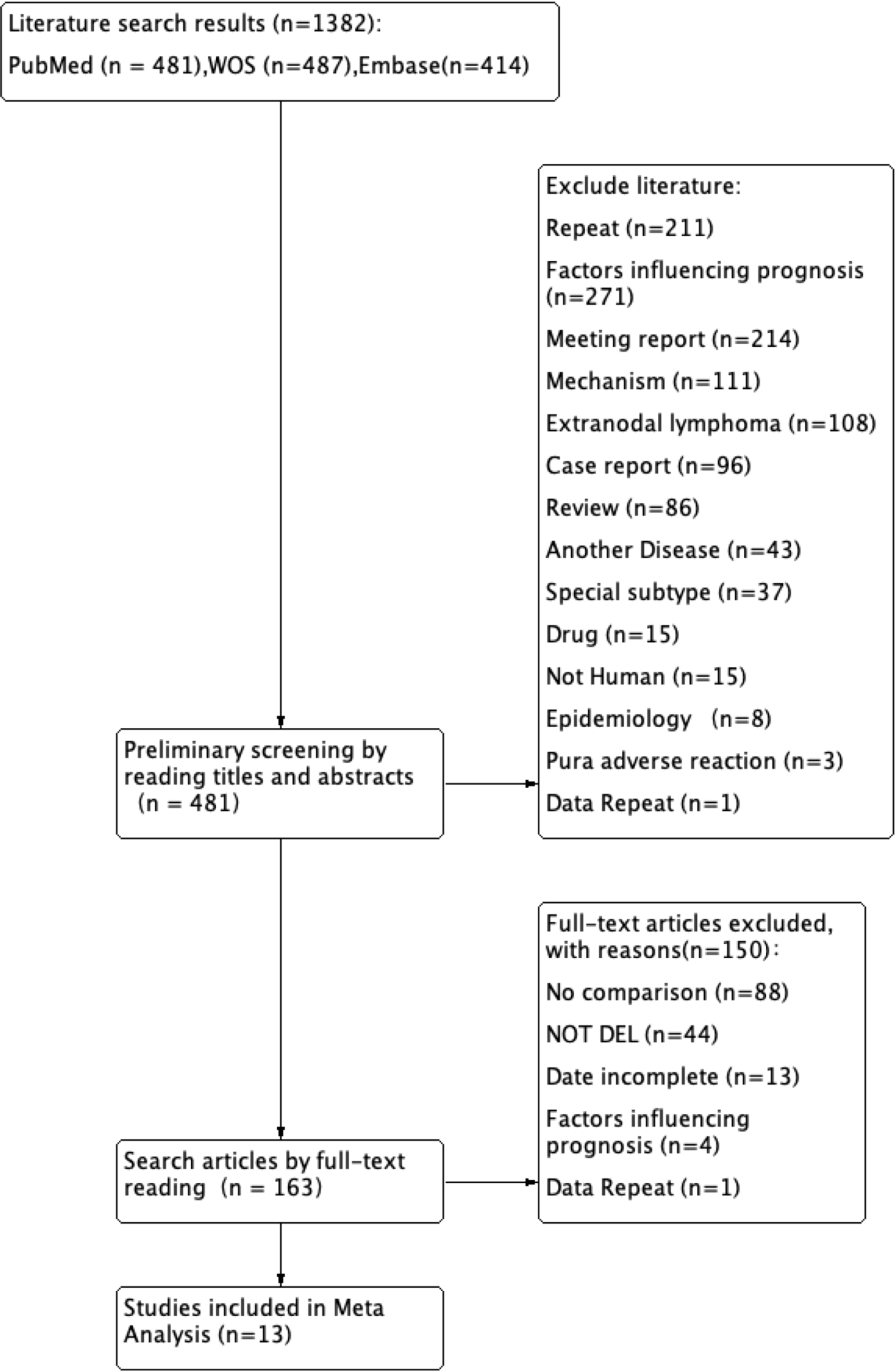
Study selection for the network meta-analysis. This PRISMA flow diagram depicts the study selection procedure for the network meta-analysis, including database searches, duplicate removal, and full-text review. PRISMA, Preferred Reporting Items for Systematic Reviews and Meta-Analyses.

Our initial search yielded 1,382 records for screening. Following a systematic deduplication process, we removed 211 records identified as duplicates. The remaining 1,171 unique records underwent title and abstract screening, resulting in 163 potentially eligible reports for full-text review. During the full-text assessment, studies were excluded based on the following pre-defined criteria: 1) not exclusively focused on the DEL population, 2) the absence of comparative efficacy data between treatments, 3) incomplete or insufficient data for analysis, 4) primary focus on prognostic factors rather than treatment outcomes, and 5) for multiple publications reporting on the same trial at different follow-up periods, only the most recent and complete report was included to avoid duplication of data. This process led to the final inclusion of 13 reports for the network meta-analysis (NMA) (**Figure 7**). These reports corresponded to 11 distinct studies (some reports were multiple publications of the same study). The evidence network encompassed six broad therapeutic classes, further detailed into eight specific treatment regimens, with a total of 1,803 patients. The detailed characteristics of the included studies are summarized in **Table 2**.

**Table 2:** Characteristics of the studies included in the network meta-analysis.

Another	Country	Research type	Years recruiting	Line	N	Trial population	Experimental group	Control group	Median age (range)	Female	Exclude DHL?	NOS
Min Zhang (77)	CHN	Cohort study	2017–2024	1	78	NE	Z+R-CHOP	R-CHOP	58 (24–76)	53.85%	N	7
Demei Feng (78)	CHN	Cohort study	2019–2024	1	213	NE	R2-CHOP/R-CHOP+BKI1	R-CHOP	58 (46–68)	41.30%	N	8
Ting Deng (79)	CHN	Cohort study	2020–2022	1	19	NE	R-CHOP+BKI2	R-CHOP	56 (32–80)	35%	N	7
Weili Zhao (80)	CHN	RCT	Mid-term		423	NE	CR-CHOP	R-CHOP	Unclear	Unclear	N	
J. S. Abramson (81)	USA	RCT	Mid-term		119	NE	Ven-R-CHOP	R-CHOP	64 (22–85)	Unclear	Y	
Peter W. M. Johnson (82)	UK	RCT	2013–2015	1	234	NE	I+R-CHOP	R-CHOP	58.6% ≥ 60y	46.30%	Y	
Jun Zhu (83)	CHN	RCT	2018–2019	1	80	NE	I+R-CHOP	R-CHOP	55% ≥ 60y	37.50%	N	
Jing Zhan (28)	CHN	Cohort study	2015–2018	1	75	NE	DA-EPOCH-R	R-CHOP	44% > 60y	51.33%	N	8
Shuhan Tang (84)	CHN	Cohort study	2016–2020	1	147	NE	DA-EPOCH-R	R-CHOP	63.5 (21–95)	50.60%	N	9
Tamer Othman (85)	USA	Cohort study	2012–2021	1	155	NE	DA-EPOCH-R	R-CHOP	65 (18–89)	43.90%	Y	6
Christopher R. D’ (86)	USA	Cohort study	2013–2016	1	90	NE	DA-EPOCH-R	R-CHOP	66	47%	N	8
A. Dodero (87)	Italy	Cohort study	2009–2017	1	114	NE	DA-EPOCH-R	R-CHOP	62 (29–81)	38%	N	8
M. ØlgodPedersen (88)	DNK	Cohort study	2004–2008	1	62	NE	DA-EPOCH-R	R-CHOP	55 (18–60)	44%	N	8

N refers to the total sample size included in the report. All the treatments were first-line therapy regimens. Some studies did not report the median age and range, instead using the proportion of cases reporting being older or younger than a certain age as reported in the study.

NE, newly diagnosed diffuse large B-cell lymphoma; R-CHOP, rituximab with cyclophosphamide, doxorubicin/liposomal doxorubicin, vincristine, and prednisone; R-CHOP+BKI, R-CHOP plus Bruton’s tyrosine kinase inhibitors (BTKis), including Z+R-CHOP, I+R-CHOP, and OR-CHOP; Z+R-CHOP, zanubrutinib plus R-CHOP; I+R-CHOP, ibrutinib plus R-CHOP; OR-CHOP, orelabrutinib plus R-CHOP; CR-CHOP, tucidinostat (chidamide) plus R-CHOP; Ven-R-CHOP, venetoclax plus R-CHOP; R2-CHOP, R-CHOP plus lenalidomide; DA-EPOCH-R, rituximab plus dose-adjusted etoposide, prednisone, vincristine, cyclophosphamide, and doxorubicin; DHL, double-hit lymphoma; NOS, Newcastle-Ottawa Scale; RCT, randomized controlled trial.

1. Demei Feng (2025) BTKi group consisted of 21 patients treated with zanubrutinib, 14 with orelabrutinib, and 1 with ibrutinib.

2. Ting Deng (2024) BTKi group consisted of 8 patients treated with zanubrutinib and 10 with orelabrutinib.

#### 3.2.2 Network geometry and consistency assessment

Thirteen studies were included in the network meta-analysis (28, 77–88), comprising nine cohort studies (including one three-arm study (78)) and four RCTs, two of which were interim analyses (80, 81).

The evidence network encompassed 14 direct treatment comparisons. The most frequent comparison was between DA-EPOCH-R and R-CHOP, informed by six studies involving 643 patients. This was followed by five studies evaluating R-CHOP+BKI (among these, two studies evaluated more than one BKI, two specifically assessed I+R-CHOP, and one assessed Z+R-CHOP). Only a single study informed each of the comparisons for R2-CHOP, CR-CHOP, and Ven-R-CHOP.

**Figure 8:**
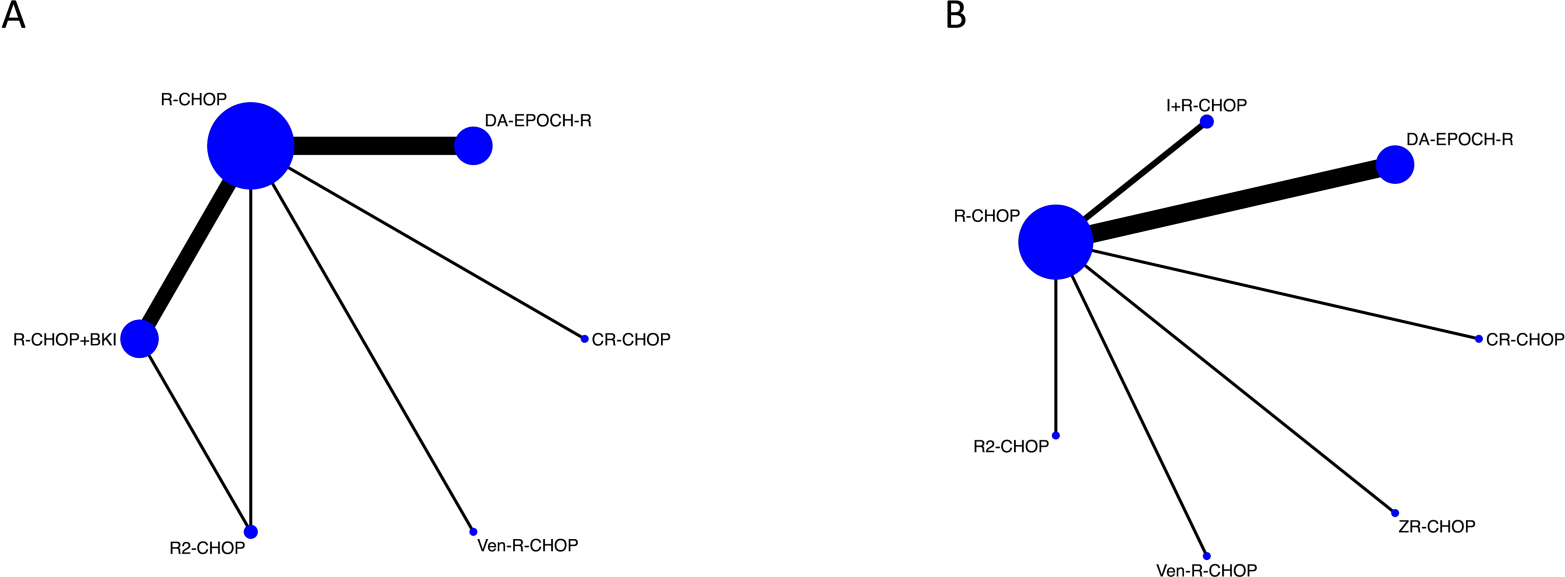
Network geometry for treatment comparisons in double-expressor lymphoma. Network relationship diagrams showing connections between treatment regimens. **(A)** Network with pooled Bruton’s tyrosine kinase inhibitor combinations. **(B)** Network with disaggregated specific Bruton’s tyrosine kinase inhibitor regimens. Node size corresponds to sample size; line thickness corresponds to number of studies. R-CHOP, rituximab plus cyclophosphamide, doxorubicin, vincristine, and prednisone; BKI, Bruton’s tyrosine kinase inhibitor; Z+R-CHOP, zanubrutinib plus R-CHOP; I+R-CHOP, ibrutinib plus R-CHOP; R2-CHOP, lenalidomide plus R-CHOP; Ven-R-CHOP, venetoclax plus R-CHOP; DA-EPOCH-R, dose-adjusted etoposide, prednisone, vincristine, cyclophosphamide, and doxorubicin plus rituximab; CR-CHOP, tucidinostat plus R-CHOP.

Using the OR as the effect measure, the global inconsistency test across the entire network was not significant (p = 0.7455), supporting the use of a consistency model for the primary analysis (**Figure 8A**). The network geometry contained a single closed loop formed by R-CHOP, R2-CHOP, and the pooled R-CHOP+BKI node. Consistency testing for this loop yielded an inconsistency factor (IF) of 0.328 (95% CI: 0.00 to 2.63). As the 95% confidence interval included zero, no significant local inconsistency was detected within this three-treatment comparison.

Given that the “R-CHOP+BKI” node encompassed distinct therapeutic agents, a secondary analysis was conducted by specifying I+R-CHOP and Z+R-CHOP as separate nodes. In this disaggregated network, one arm from a three-arm study was excluded due to its evaluation of a mixed BKI category. This resulted in a network structure with no closed loops, precluding loop-specific inconsistency testing. The global inconsistency test for this network was also non-significant (p = 0.0715), and a consistency model was similarly applied for analysis (**Figure 8B**).

#### 3.2.3 Network meta-analysis results: efficacy rankings and pairwise comparisons

**Figure 9:**
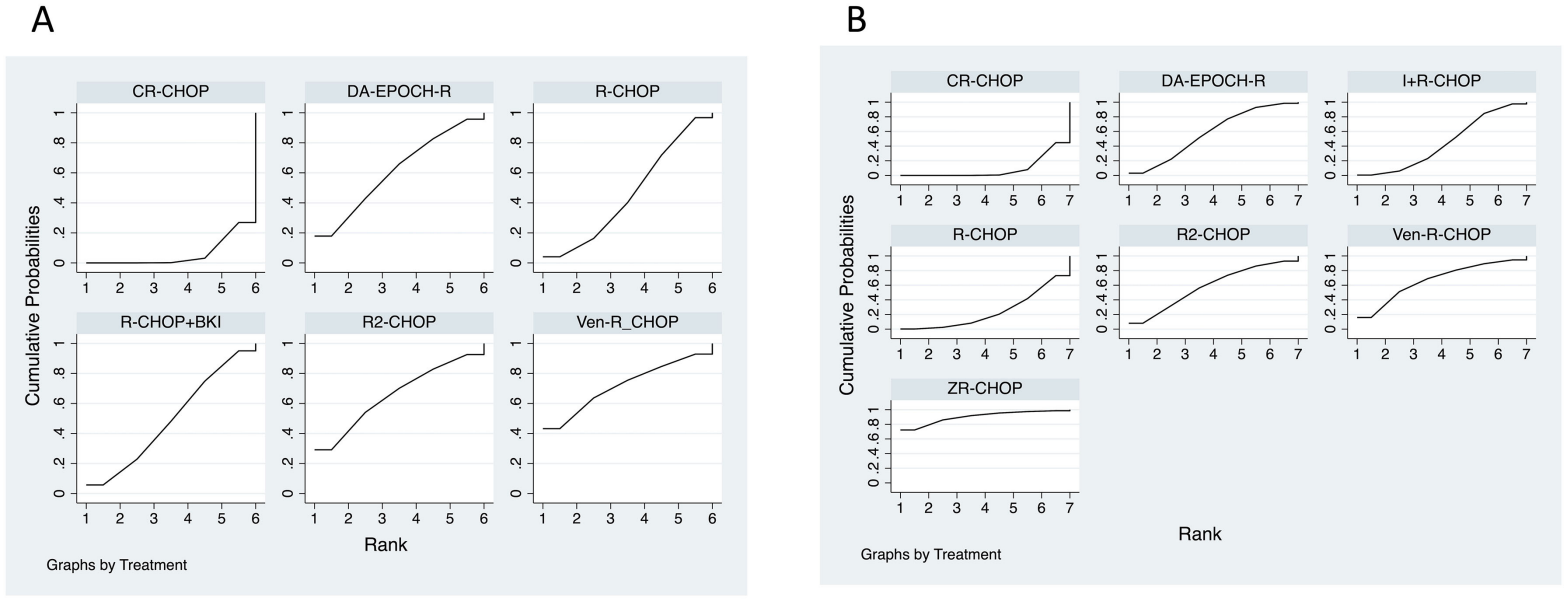
Cumulative ranking curves for treatment efficacy in double-expressor lymphoma. Surface under the cumulative ranking curve (SUCRA) plots showing probability of being the best treatment. **(A)** Analysis with pooled Bruton’s tyrosine kinase inhibitor combinations. **(B)** Analysis with disaggregated specific Bruton’s tyrosine kinase inhibitor regimens. SUCRA, surface under the cumulative ranking curve; R-CHOP, rituximab plus cyclophosphamide, doxorubicin, vincristine, and prednisone; other abbreviations as in **Figure 8**.

**Figure 10:**
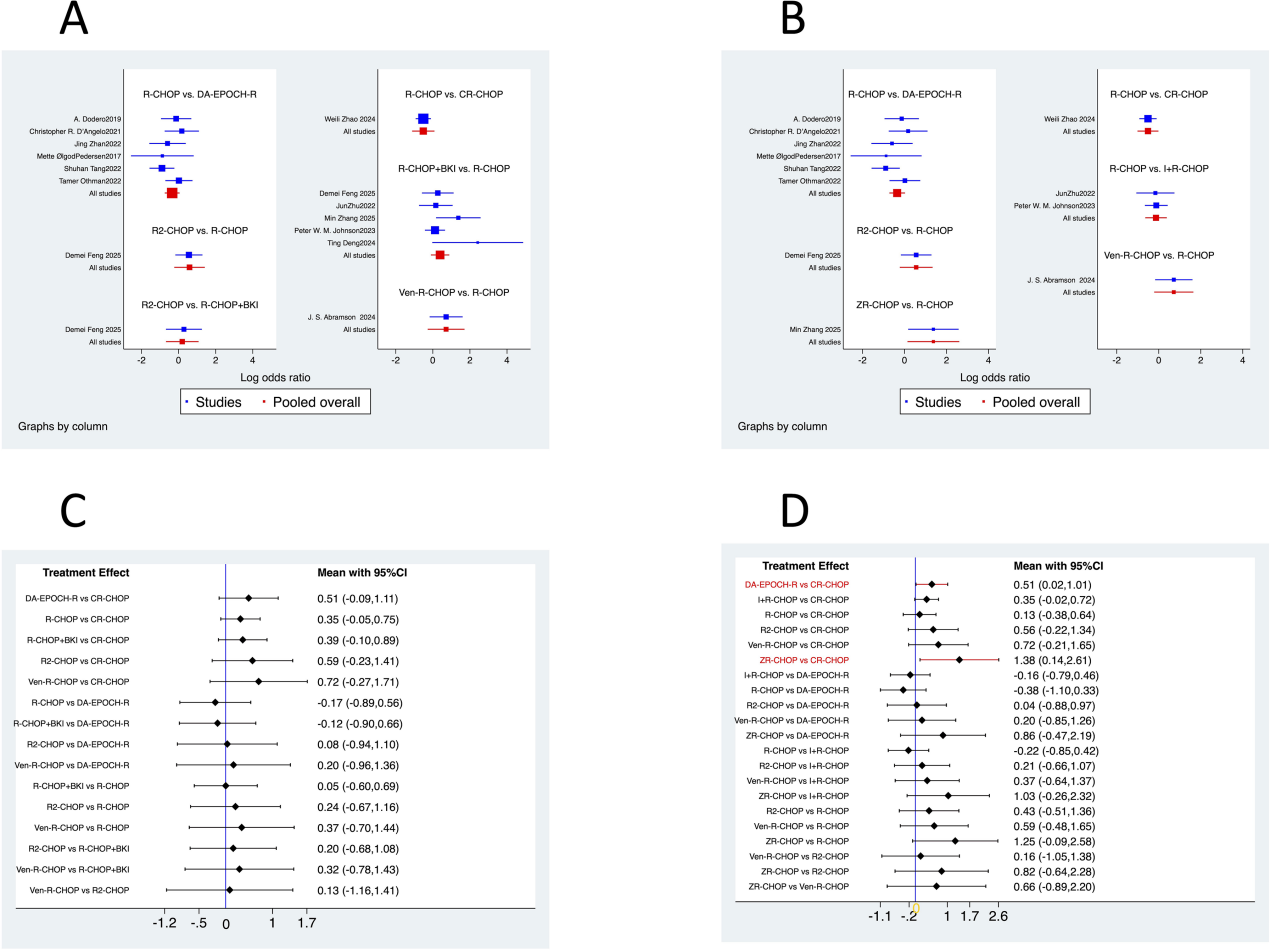
Relative treatment effects and pairwise comparisons in the network meta-analysis. Forest plots showing odds ratios for complete response rates. **(A)** All treatment comparisons (pooled network). **(B)** All treatment comparisons (disaggregated network). **(C)** Key pairwise comparisons (pooled network). **(D)** Key pairwise comparisons (disaggregated network). OR, odds ratio; CI, confidence interval; other abbreviations as in **Figure 8**.

**Figure 11:**
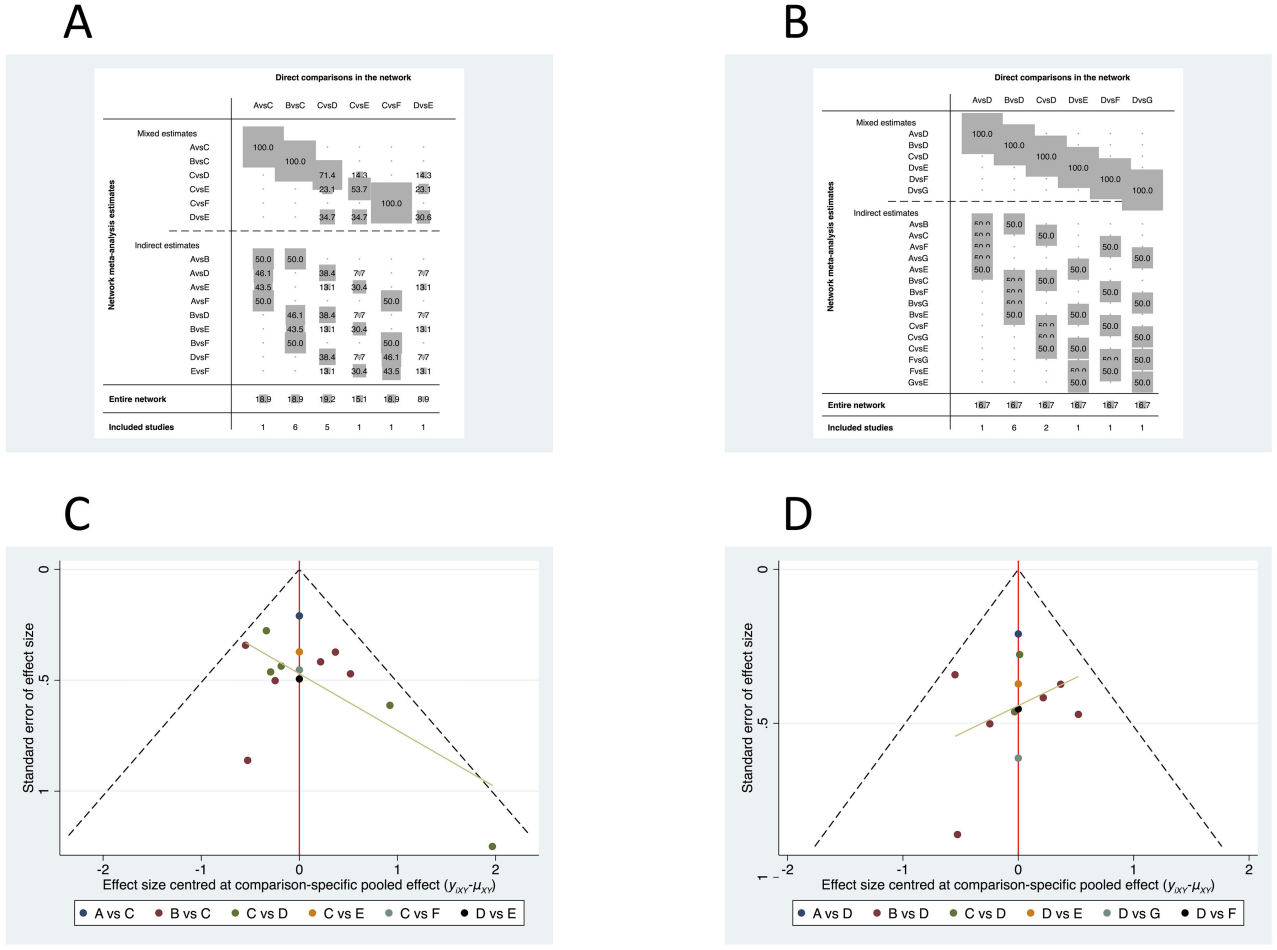
Contribution plots and funnel plots for network meta-analysis. **(A)** Contribution plot for pooled network. **(B)** Contribution plot for disaggregated network. **(C)** Funnel plot for pooled network. **(D)** Funnel plot for disaggregated network. Contribution plots show percentage contribution of each study to network estimates; funnel plots assess publication bias.

Analysis with BKIs pooled: The SUCRA values, which estimate the probability of a treatment being the best, ranked the regimens as follows when all BKI-based combinations were pooled: Ven-R-CHOP (SUCRA = 72.0%) > R2-CHOP (65.8%) > DA-EPOCH-R (61.0%) > R-CHOP+BKI (49.3%) > R-CHOP (45.8%) > CR-CHOP (6.0%). This ranking is visually represented in the cumulative ranking plot (**Figure 9A**) and the forest plot of relative effects (**Figure 10A**). A league table presenting the OR and 95% CI for every possible pairwise comparison was generated (**Table 3A**, with columns as the experimental group and rows as the control group). However, none of these direct or indirect pairwise comparisons within this network reached statistical significance (**Figure 10C**, **Table 3A**). A network contribution plot detailing the percentage of information each study contributed to the various treatment comparisons was generated (**Figure 11A**).

**Table 3A:** League table of different treatment regimens (total network).

Ven-R-CHOP					
1.13 (0.31, 4.11)	R2-CHOP				
1.23 (0.38, 3.91)	1.08 (0.39, 2.99)	DA-EPOCH-R			
1.38 (0.46, 4.19)	1.22 (0.51, 2.94)	1.13 (0.52, 2.46)	R-CHOP+BKI		
1.45 (0.50, 4.22)	1.28 (0.51, 3.19)	1.18 (0.57, 2.43)	1.05 (0.55, 1.99)	R-CHOP	
2.05 (0.76, 5.52)	1.81 (0.79, 4.10)	1.67 (0.92, 3.05)	1.48 (0.90, 2.43)	1.41 (0.95, 2.11)	CR-CHOP

The numbers in the grid represent the odds ratios (95% CI) comparing the two treatment regimens corresponding to each row and column, with the column representing the experimental group and the row representing the control group.

Ven-R-CHOP, R-CHOP plus venetoclax; R2-CHOP, R-CHOP plus lenalidomide; DA-EPOCH-R, dose-adjusted etoposide, prednisone, vincristine, cyclophosphamide, and doxorubicin plus rituximab; R-CHOP+BKI, R-CHOP plus other Bruton’s tyrosine kinase inhibitors; R-CHOP, rituximab with cyclophosphamide, doxorubicin, vincristine, and prednisone; CR-CHOP, tucidinostat (chidamide) plus R-CHOP.

The yellow sections indicate specific treatment regimens, and the green sections show pairwise comparisons of ORs with 95% confidence intervals.

Analysis with specific BKI regimens: To rank individual BKI-based therapies, a secondary NMA was performed with I+R-CHOP and Z+R-CHOP as separate nodes. The SUCRA ranking from this analysis was as follows: Z+R-CHOP (90.8%) > Ven-R-CHOP (66.4%) > DA-EPOCH-R (58.3%) > R2-CHOP (58.1%) > I+R-CHOP (43.8%) > R-CHOP (23.7%) > CR-CHOP (9.0%). This ranking is visualized in the corresponding cumulative ranking plot (**Figure 9B**) and forest plot (**Figure 10B**), with detailed pairwise ORs presented in a league table (**Figure 11B**, **Table 3B**). Notably, in this analysis, two pairwise comparisons yielded statistically significant odds ratios: Z+R-CHOP vs. CR-CHOP [OR = 3.96, 95% CI: (1.15, 13.59)] and DA-EPOCH-R vs. CR-CHOP [OR = 1.67, 95% CI: (1.02, 2.75)] (**Figure 10D**). These significant findings confirm the superiority of Z+R-CHOP and DA-EPOCH-R over CR-CHOP, aligning with their high SUCRA rankings. The contribution plot for this analysis is presented in **Figure 11B**.

**Table 3B:** League table of different treatment regimens (disaggregated network).

Z+R-CHOP							
1.93 (0.41, 9.07)	Ven-R-CHOP						
2.37 (0.63, 8.96)	1.23 (0.43, 3.53)	DA-EPOCH-R					
2.27 (0.53, 9.78)	1.17 (0.35, 3.96)	0.96 (0.38, 2.42)	R2-CHOP				
2.79 (0.77, 10.13)	1.44 (0.53, 3.94)	1.18 (0.63, 2.19)	1.23 (0.52, 2.92)	I+R-CHOP			
3.48 (0.91, 13.23)	1.80 (0.62, 5.21)	1.47 (0.72, 3.00)	1.53 (0.60, 3.90)	1.25 (0.66, 2.35)	R-CHOP	
3.96 (1.15, 13.59)	2.05 (0.81, 5.20)	1.67 (1.02, 2.75)	1.74 (0.80, 3.81)	1.42 (0.98, 2.06)	1.14 (0.68, 1.90)	CR-CHOP

The numbers in the grid represent the odds ratios (95% CI) comparing the two treatment regimens corresponding to each row and column, with the column representing the experimental group and the row representing the control group.

Z+R-CHOP, zanubrutinib plus R-CHOP; Ven-R-CHOP, R-CHOP plus venetoclax; DA-EPOCH-R, dose-adjusted etoposide, prednisone, vincristine, cyclophosphamide, and doxorubicin plus rituximab; R2-CHOP, R-CHOP plus lenalidomide; I+R-CHOP, R-CHOP plus ibrutinib; R-CHOP, rituximab with cyclophosphamide, doxorubicin, vincristine, and prednisone; CR-CHOP, tucidinostat (chidamide) plus R-CHOP.

The orange-red sections represent treatment regimens, and the purple sections display pairwise ORs with 95% CIs.

It is important to note that some studies have reported (21) CR-CHOP to be superior to R-CHOP. Chidamide, a histone deacetylase (HDAC) inhibitor, has demonstrated the ability to overcome rituximab-induced CD20 downregulation, synergize with rituximab-mediated cytotoxicity, and potentially overcome acquired chemoresistance. This body of evidence suggests that the efficacy of CR-CHOP is likely stronger than predicted by its low SUCRA value in our analysis. The observed discrepancy may be attributed to limitations inherent in the network geometry, such as the peripheral position of the CR-CHOP node and its reliance on long indirect evidence chains for estimation, which can introduce imprecision. Alternatively, heterogeneity among studies evaluating CR-CHOP could be a contributing factor. Therefore, the definitive efficacy of CR-CHOP in the DEL population warrants further investigation.

#### 3.2.4 Assessment of publication bias

Funnel plot analysis was performed to assess potential publication bias. The plots for both the primary network (with pooled BKI) and the secondary network (with specific BKI regimens) demonstrated approximate symmetry (**Figures 11C, D**). This visual inspection suggests no substantial evidence of publication bias across the studies included in either network meta-analysis.

#### 3.2.5 Safety analysis

Among the 13 included studies, seven reported data on treatment-emergent adverse events (AEs) (28, 77–79, 81, 83). Four studies compared AEs between R-CHOP+BKI and standard R-CHOP. These four comprised two studies investigating regimens with more than one BKI agent, one study using Z+R-CHOP, and one study using I+R-CHOP (**Table 4A**). For other treatment comparisons versus R-CHOP, one study each was available for R2-CHOP, DA-EPOCH-R, and Ven-R-CHOP (**Table 4B**).

**Table 4A:** Adverse reactions (R-CHOP+BKI vs. R-CHOP).

	Z+R-CHOP vs. R-CHOP		I+R-CHOP vs. R-CHOP		Demei Feng (2025) (2)		Ting Deng (2024)	
	OR	p	OR	p	OR	p	OR	p
Hematological (Grade 3–4)								
Anemia	1.02 (0.39, 2.71)	0.9644	–	–	0.88 (0.27, 2.85)	0.8246	1.13 (0.30, 4.24)	0.962
Thrombocytopenia	0.42 (0.15, 1.19)	0.1017	–	–	1.95 (0.60, 6.31)	0.2641	0.91 (0.23, 3.52)	0.89
Neutropenia	0.54 (0.20, 1.44)	0.2172	0.76 (0.43, 1.35)	0.3450	0.60 (0.26, 1.31)	0.1924	1.14 (0.30, 4.37)	0.845
Febrile neutropenia	1.14 (0.37, 3.49)	0.8182	1.71 (0.83, 3.54)	0.1488	–	–	0.65 (0.12, 3.46)	0.613
Leukopenia	–	–	0.94 (0.53, 1.67)	0.8250	0.69 (0.32, 1.47)	0.3366	–	–
Non-hematological adverse events								
Infection	1.43 (0.52, 3.96)	0.4835	–	–	0.24 (0.06, 0.94)	0.0412	0.52 (0.12, 2.33)	0.395
Atrial fibrillation	0.77 (0.03, 19.68)	0.8761	3.17 (0.54, 18.66)	0.2017	–	–	1.50 (0.22, 10.31)	0.68
Hemorrhage/ecchymosis	0.58 (0.06, 5.49)	0.6343	–	–	–	–	–	–
Hyperuricemia	0.40 (0.12, 1.34)	0.1375	–	–	–	–	–	–
Elevated transaminases	0.57 (0.19, 1.68)	0.3089	–	–	1.29 (0.59, 2.80)	0.5209	0.45 (0.12, 1.72)	0.242
Nausea and vomiting	0.39 (0.13, 1.15)	0.0879	–	–	0.60 (0.03, 12.90)	0.7479	–	–
Anorexia	0.79 (0.26, 2.35)	0.6706	–	–	–	–	–	–
Diarrhea	0.49 (0.10, 2.45)	0.3828	–	–	0.60 (0.03, 12.90)	0.7479	2.88 (0.48, 17.45)	0.249
Fatigue	0.38 (0.14, 1.05)	0.0632	–	–	–	–	–	–
Elevated bilirubin	–	–	–	–	3.45 (0.94, 12.70)	0.0623	–	–
Decreased albumin	–	–	–	–	1.01 (0.45, 2.28)	0.982	–	–
Rash	–	–	–	–	3.38 (0.80, 14.26)	0.098	–	–
Sensory neuropathy	–	–	–	–	0.69 (0.19, 2.58)	0.5838	–	–
Oral ulcers	–	–	–	–	0.77 (0.08, 7.13)	0.8191	–	–
Sepsis	–	–	–	–	–	–	–	–

R-CHOP+BKI, R-CHOP plus other Bruton’s tyrosine kinase inhibitors; R-CHOP, rituximab with cyclophosphamide, doxorubicin, vincristine, and prednisone; Z+R-CHOP, zanubrutinib plus R-CHOP; I+R-CHOP, R-CHOP plus ibrutinib.

**Table 4B:** Adverse reactions.

	R2-CHOP vs. R-CHOP		DA-EPOCH-R vs. R-CHOP		Ven-R-CHOP vs. R-CHOP		R-CHOP+BKI vs. R-CHOP		
	OR	p	OR	p	OR	p	OR	p	I^2^
Hematological (Grade 3–4)									
Anemia	0.22 (0.05, 1.01)	0.0517	2.23 (0.81, 6.16)	0.1222	9.64 (2.09, 44.51)	0.0037	1.00 (0.52, 1.92)	0.96	0
Thrombocytopenia	2.08 (0.86, 5.03)	0.1043	2.89 (1.17, 7.14)	0.0216	5.75 (1.55, 21.32)	0.0089	0.88 (0.36, 2.17)	0.15	47%
Neutropenia	1.38 (0.74, 2.54)	0.3091	3.04 (1.50, 6.17)	0.0021	1.62 (0.76, 3.44)	0.2101	0.69 (0.46, 1.04)	0.79	0
Febrile neutropenia	–	–	1.52 (0.65, 3.52)	0.3328	2.43 (0.70, 8.43)	0.1595	1.37 (0.77, 2.46)	0.54	0
Leukopenia	1.00 (0.54, 1.83)	0.9873					0.84 (0.53, 1.33)	0.53	0
Non-hematological adverse events									
Infection	0.13 (0.02, 1.03)	0.0529	–	–	–	–	0.68 (0.20, 2.36)	0.16	46%
Atrial fibrillation	–	–	–	–	–	–	1.88 (0.50, 7.08)	0.69	0
Hemorrhage/ecchymosis	–	–	–	–	–	–	–	–	–
Hyperuricemia	–	–	–	–	–	–	–	–	–
Elevated transaminases	1.35 (0.72, 2.55)	0.3515	–	–	–	–	0.81 (0.42, 1.57)	0.29	20%
Nausea and vomiting	0.86 (0.08, 9.67)	0.9023	–	–	–	–	0.41 (0.15, 1.13)	0.79	0
Anorexia	–	–	–	–	–	–	–	–	–
Diarrhea	1.75 (0.24, 12.70)	0.5820	–	–	–	–	1.00 (0.30, 3.29)	0.33	9%
Fatigue	–	–	–	–	14.26 (0.78, 259.49)	0.0726	–	–	–
Elevated bilirubin	1.78 (0.50, 6.41)	0.3755	–	–	–	–	–	–	–
Decreased albumin	0.57 (0.28, 1.19)	0.1351	–	–	–	–	–	–	–
Rash	3.79 (1.09, 13.13)	0.0356	–	–	–	–	–	–	–
Sensory neuropathy	1.22 (0.49, 3.04)	0.6638	–	–	–	–	–	–	–
Oral ulcers	1.31 (0.28, 6.03)	0.7319	–	–	–	–	–	–	–
Sepsis	–	–	–	–	–	–	–	–	–

R2-CHOP, R-CHOP plus lenalidomide; R-CHOP, rituximab with cyclophosphamide, doxorubicin, vincristine, and prednisone; DA-EPOCH-R, dose-adjusted etoposide, prednisone, vincristine, cyclophosphamide, and doxorubicin plus rituximab; Ven-R-CHOP, R-CHOP plus venetoclax; R-CHOP+BKI, R-CHOP plus other Bruton’s tyrosine kinase inhibitors.

Safety outcomes were evaluated using ORs as the effect measure, calculating the OR, 95% CI, and p-value for reported AEs. For the four R-CHOP+BKI vs. R-CHOP studies, if an AE type was reported in two or more studies, a meta-analysis was performed, and the I^2^ statistic was calculated to assess heterogeneity. For AE types reported in only a single study, the OR, 95% CI, and p-value from that study were presented individually.

The following comparisons showed statistically significant differences: DA-EPOCH-R was associated with a significantly higher incidence of Grade 3–4 thrombocytopenia [OR = 2.89 (95% CI: 1.17, 7.14), p = 0.0216] and Grade 3–4 neutropenia [OR = 3.04 (95% CI: 1.50, 6.17), p = 0.0021] compared to R-CHOP.

R2-CHOP resulted in more frequent rash [OR = 3.79 (95% CI: 1.09, 13.13), p = 0.0356] than R-CHOP.

Ven-R-CHOP showed a significantly higher incidence of Grade 3–4 anemia [OR = 9.64 (95% CI: 2.09, 44.51), p = 0.0037] and Grade 3–4 thrombocytopenia [OR = 5.75 (95% CI: 1.55, 21.32), p = 0.0089] compared to R-CHOP.

Notably, a study involving a specific triple-BKI regimen reported a significantly lower incidence of infection compared to R-CHOP [OR = 0.24 (95% CI: 0.06, 0.94), p = 0.0412].

Furthermore, Grade 5 (fatal) AEs were reported in the Ven-R-CHOP study, with the following causality assessments: pulmonary infection (possibly related), sudden death of unknown cause (possibly related), and respiratory failure (unlikely to be related). Combined with its significant hematological toxicity profile, this underscores the critical need for vigilant safety monitoring when using the Ven-R-CHOP regimen.

In summary, the available safety data suggest that most novel or intensified treatment strategies for DEL are associated with an increased incidence of specific AEs compared to standard R-CHOP. BKI-containing regimens, particularly certain specific combinations, may offer a potentially more favorable safety profile. However, this observation is tempered by a significant limitation: the overall number of comparative safety studies is low, and many individual comparisons did not reach statistical significance. Therefore, the safety conclusions, especially regarding BKI-based therapies, require validation in larger, prospectively designed studies.

## 4 Discussion

### 4.1 Key finding

The detrimental impact of the double-expressor (DEL) phenotype on survival in DLBCL is widely acknowledged in clinical practice (71). However, quantitative estimates of its effect size on patient survival and prognosis have been lacking, as has a clear comparison of its prognostic impact between newly diagnosed and R/R disease settings.

This study first addressed the prognostic significance of DEL in DLBCL by conducting a meta-analysis of all relevant studies. The results conclusively demonstrate that DEL is an independent adverse prognostic factor. For PFS, the pooled HR was 1.78 (95% CI: 1.50–2.10, p < 0.00001), with HRs of 1.74 (95% CI: 1.45–2.08, p < 0.00001) in newly diagnosed patients and 1.97 (95% CI: 1.21–3.19, p = 0.006) in R/R patients. For OS, the pooled HR was 1.90 (95% CI: 1.68–2.15, p < 0.00001), with HRs of 1.90 (95% CI: 1.65–2.18, p < 0.0001) and 1.94 (95% CI: 1.48–2.54, p < 0.00001) in the newly diagnosed and R/R groups, respectively. Although the point estimates for R/R disease appear higher, the overlapping confidence intervals preclude a definitive conclusion that DEL confers a greater risk in this population compared to newly diagnosed patients. This observation warrants investigation in larger, specifically designed cohorts.

With the recognition of DEL as a distinct biologic subtype (4), it is imperative to define the prognostic role of established clinical factors specifically within this entity. Our meta-analysis evaluated factors with data from at least two studies, including TP53, IPI, DHL, age, gender, ECOG performance status, Ann Arbor stage, extranodal involvement, LDH, B symptoms, cell of origin (COO), and Ki-67. Within the DEL population, the following were identified as statistically significant independent prognostic factors: for PFS, high IPI (HR = 2.01, 95% CI: 1.36–2.97, p = 0.0005), advanced age (HR = 1.47, 95% CI: 1.11–1.95, p = 0.007), advanced Ann Arbor stage (HR = 2.37, 95% CI: 1.40–3.99, p = 0.0001), elevated LDH (HR = 1.74, 95% CI: 1.29–2.36, p = 0.0003), and the presence of B symptoms (HR = 2.13, 95% CI: 1.37–3.32, p = 0.008); for OS, TP53 mutation (HR = 2.52, 95% CI: 1.07–5.97, p = 0.04), high IPI (HR = 2.58, 95% CI: 1.41–4.75, p = 0.002), advanced age (HR = 1.70, 95% CI: 1.27–2.27, p = 0.0004), poor ECOG status (HR = 1.86, 95% CI: 1.06–3.27, p = 0.03), advanced Ann Arbor stage (HR = 2.61, 95% CI: 1.71–3.98, p < 0.0001), elevated LDH (HR = 2.10, 95% CI: 1.09–4.07, p = 0.03), and the presence of B symptoms (HR = 2.02, 95% CI: 1.20–3.39, p = 0.008).

Given the poorer prognosis associated with DEL, determining the optimal therapeutic strategy is crucial. A network meta-analysis of available comparative studies was performed, evaluating regimens including DA-EPOCH-R, R2-CHOP, R-CHOP+BKI (encompassing zanubrutinib, ibrutinib, and unspecified combinations), CR-CHOP, and Ven-R-CHOP, all compared against standard R-CHOP. Based on SUCRA rankings, Z+R-CHOP and Ven-R-CHOP emerged as the most efficacious options. An intriguing finding emerged with CR-CHOP: its theoretically promising profile—based on the mechanistic rationale of the HDAC inhibitor chidamide to overcome drug resistance—contrasted with its low ranking in our network meta-analysis. Consequently, while its present SUCRA score is low, its potential efficacy warrants dedicated evaluation in larger, prospective studies designed for this subtype. Regarding safety, intensified regimens like R2-CHOP, Ven-R-CHOP, and DA-EPOCH-R were associated with a higher burden of adverse events compared to R-CHOP. In contrast, BKI-based combinations, particularly zanubrutinib, showed a trend toward a more favorable safety profile, although this finding also necessitates confirmation in larger prospective trials.

### 4.2 Discussion

While this study elucidates several key prognostic factors within the DEL subtype, it does not provide a complete picture. Several established and emerging factors in DLBCL remain to be validated specifically in DEL. For instance, the prognostic role of bulky disease, a common consideration in DLBCL, could not be assessed due to a lack of eligible studies. Furthermore, novel factors such as elevated leptin levels—which may promote lymphomagenesis and progression by fostering a pro-inflammatory immune microenvironment (89)—and metabolic parameters like maximum standardized uptake value (SUVmax) on positron emission tomography–computed tomography (PET–CT), a recognized independent prognostic factor in DLBCL (90, 91), warrant investigation in DEL. Intriguingly, the “obesity paradox” observed in some cancers, which has also been identified in DLBCL, suggests that obese patients may experience better treatment response and survival, potentially mediated by higher vitamin D and CD8+ NK cell levels (92).

Precise prognostic assessment in DEL necessitates a mechanistic understanding of disease pathogenesis. Beyond clinical and metabolic parameters, epigenetic features are emerging as a critical dimension in DEL prognostic stratification. Aberrant epigenetic mechanisms—including DNA methylation heterogeneity and specific miRNA expression profiles (e.g., elevated miR-21 and miR-155 expression)—have been demonstrated to exert a significant prognostic impact (93).

This highlights the complexity of prognostic modeling. Therefore, validating the impact of both established DLBCL factors and newly discovered biomarkers within the DEL subtype requires large-scale, dedicated datasets.

The therapeutic landscape for DEL is rapidly evolving, but our analysis is constrained by the inherent lag in publication and the fact that many clinical trials have not stratified results specifically for this newly defined entity. Consequently, several promising or established regimens were not included in our network meta-analysis. For example, polatuzumab vedotin (Pola), an anti-CD79b antibody–drug conjugate (ADC), combined with R-CHP (Pola-R-CHP), has emerged as a first-line treatment for DLBCL and has been demonstrated to significantly improve PFS in DEL, suggesting its potential to overcome the relative resistance of this subtype to conventional R-CHOP (94). The combination of zanubrutinib, lenalidomide, and R-CHOP (ZR2-CHOP), which has shown progression-free survival benefit in DEL (13), was not evaluated in comparative studies eligible for our analysis. Beyond combination chemotherapies, mechanistic research points to other rational therapeutic avenues. For instance, the epigenetic-targeted therapeutic strategies discussed above warrant further consideration. Bromodomain and extraterminal domain (BET) inhibitors can rapidly downregulate MYC and BCL2 transcription and remodel the immune microenvironment. Their synergy with HDAC inhibitors may simultaneously target MYC-driven proliferation and BCL2-mediated anti-apoptosis, potentially triggering Poly(ADP-ribose) Polymerase (PARP)-mediated cell death. Furthermore, combining BET inhibitors with venetoclax may overcome primary resistance, making BET+HDAC+BCL2 inhibitor combinations a promising frontier for DEL therapy (95). Similarly, preclinical and clinical evidence suggest that chidamide (an HDAC inhibitor) combined with the Dexamethasone, Ifosfamide, Cisplatin, Etoposide (DICE) regimen can downregulate MYC and BCL2 expression, offering a viable option for DEL and potentially reversing drug resistance in relapsed/refractory disease (9). Additionally, agents with preclinical promise, such as acyclic terpenoids targeting BCL2, have demonstrated anti-lymphoma activity in murine models and human lymphoma cell lines (96), indicating another potential direction for future drug development.

### 4.3 Limitations

This study has several important limitations. For the prognostic meta-analysis, inherent heterogeneity exists across the included studies regarding patient baseline characteristics and treatment strategies, a common challenge in such syntheses. More critically, the number of studies investigating specific prognostic factors within DEL is limited, leading to insufficient sample size for several comparisons. The absence of studies on factors like bulky disease may introduce bias. For the network meta-analysis, the limited number of studies, with many comparisons informed by only a single trial, increases uncertainty in the efficacy rankings. The safety analysis is particularly constrained by the sparse and inconsistent reporting of adverse events across studies, necessitating cautious interpretation and further validation to conclusively establish the safety profiles of these regimens. These limitations underscore the need for future research to prioritize dedicated, well-powered studies within the DEL population, employing standardized reporting for both outcomes and toxicities.

## 5 Conclusion

This study provides a comprehensive and systematic evidence synthesis that integrates prognostic and therapeutic comparisons specifically for the DEL subtype. We have precisely quantified the significant prognostic detriment associated with DEL, identified key adverse prognostic factors operating within this distinct entity, and evaluated the comparative efficacy and safety of contemporary first-line treatment strategies. Notably, Bruton’s tyrosine kinase inhibitor (BKI)-based regimens, particularly zanubrutinib in combination with R-CHOP, not only emerged among the most efficacious approaches but also were associated with a more favorable hematological toxicity profile. These findings underscore the necessity of recognizing DEL as a distinct clinicobiological subtype in clinical practice. Future research and trial design should prioritize direct comparisons among novel regimens within this high-risk population while rigorously monitoring their long-term safety.

## Data Availability

The original contributions presented in the study are included in the article/**Supplementary Material**. Further inquiries can be directed to the corresponding author.
