# Supplementary material for: A systematic evaluation of double-expressor lymphoma: prognostic impact, determinants of outcome, and comparative efficacy and safety of novel therapies
Source: Front Oncol. 2026 May 21;16:1823300. doi: 10.3389/fonc.2026.1823300 (PMC13233246; doi:10.3389/fonc.2026.1823300)
Supplement: Supplementary file 1 [file Table1.docx]

Table S1A Search strategy of PubMed

| #1 | Lymphoma, Large B-Cell, Diffuse [Title/Abstract] |
| --- | --- |
| #2 | Diffuse Large B-Cell Lymphoma [Title/Abstract] |
| #3 | Diffuse Large B Cell Lymphoma [Title/Abstract] |
| #4 | Lymphoma, Histiocytic [Title/Abstract] |
| #5 | Histiocytic Lymphomas [Title/Abstract] |
| #6 | Lymphoma, Large Lymphoid, Diffuse [Title/Abstract] |
| #7 | Lymphoma, Histiocytic, Diffuse [Title/Abstract] |
| #8 | Lymphoma, Large Cell, Diffuse [Title/Abstract] |
| #9 | Lymphoma, Large-Cell, Diffuse [Title/Abstract] |
| #10 | Diffuse Large-Cell Lymphoma [Title/Abstract] |
| #11 | Diffuse Large Cell Lymphoma [Title/Abstract] |
| #12 | Diffuse Large-Cell Lymphomas [Title/Abstract] |
| #13 | Diffuse, Large B-Cell, Lymphoma [Title/Abstract] |
| #14 | Histiocytic Lymphoma [Title/Abstract] |
| #15 | Histiocytic Lymphoma, Diffuse [Title/Abstract] |
| #16 | Diffuse Histiocytic Lymphoma [Title/Abstract] |
| #17 | Diffuse Histiocytic Lymphomas [Title/Abstract |
| #18 | Lymphoma, Diffuse Histiocytic [Title/Abstract] |
| #19 | Large Lymphoid Lymphoma, Diffuse [Title/Abstract] |
| #20 | Large-Cell Lymphoma, Diffuse [Title/Abstract] |
| #21 | Large Cell Lymphoma, Diffuse [Title/Abstract] |
| #22 | Lymphoma, Diffuse Large-Cell [Title/Abstract] |
| #23 | Lymphoma, Diffuse Large Cell [Title/Abstract] |
| #24 | #1 OR #2 OR #3 OR #4 OR #5 OR #6 OR #7 OR #8 OR #9 OR #10 OR #11 OR #12 OR #13 OR #14 OR #15 OR #16 OR #17 OR #18 OR #19 OR #20 OR #21 OR #22 OR #23 |
| #25 | double expressor lymphoma |
| #26 | MYC/BCL-2 co-expression |
| #27 | co-expression of MYC and BCL2 protein |
| #28 | MYC/BCL2 double-expression |
| #29 | coexpressing BCL2 and MYC |
| #30 | “DEL” |
| #31 | #25 OR #26 OR #27 OR #28 OR #29 OR #30 |
| #32 | #24 AND #31 |

Table S1B Search strategy of Embase

| #1 | 'c-myc'/de |
| --- | --- |
| #2 | 'bcl2'/de |
| #3 | #1 AND #2 |
| #4 | (myc NEAR/2 bcl2):ti,ab,kw |
| #5 | (double NEAR/3 express):ti,ab,kw |
| #6 | 'de dlbcl':ti,ab,kw |
| #7 | coexpress:ti,ab,kw |
| #8 | #3 OR #4 OR #5 OR #6 OR #7 |
| #9 | 'diffuse large b cell lymphoma'/de |
| #10 | 'dlbcl':ti,ab,kw |
| #11 | #9 OR #10 |
| #12 | #8 AND #11 |
| #13 | 'double hit':ti,ab,kw |
| #14 | dh:ti,ab,kw |
| #15 | 'triple hit':ti,ab,kw |
| #16 | #13 OR #14 OR #15 |
| #17 | #12 NOT #16 |
| #18 | [2010-2025]/py |
| #19 | #17 AND #18 |
| #20 | 'article'/it |
| #21 | 'clinical trial'/it |
| #22 | #20 OR #21 |
| #23 | #19 AND #22 |

Table S1C Search strategy of “Web of Science”

| #1 | MYC |
| --- | --- |
| #2 | "c-Myc" |
| #3 | MYCN |
| #4 | #1 OR #2 OR #3 |
| #5 | BCL2 |
| #6 | "B-cell lymphoma 2 |
| #7 | #6 OR #7 |
| #8 | "co-expression" |
| #9 | "coexpress*" |
| #10 | "concurrent*" |
| #11 | "simultaneous*" |
| #12 | "dual expression" |
| #13 | "both positive" |
| #14 | #8 OR #9 OR #10 OR #11 OR #12 OR #13 OR #14 |
| #15 | #4 AND #7 AND #14 |
| #16 | "double expressor" |
| #17 | "double-expressor" |
| #18 | "DE-DLBCL" |
| #19 | "dual expressor" |
| #20 | #16 OR #17 OR #18 OR #19 |
| #21 | #15 OR #20 |
| #22 | "diffuse large B-cell lymphoma" |
| #23 | "DLBCL" |
| #24 | #22 OR #23 |
| #25 | #21 AND #24 |
| #26 | TS=#25 |

Table S2A Sample size for DEL vs non-DEL

| Another | Year | N | N(DEL) | N(non-DEL) |
| --- | --- | --- | --- | --- |
| Gayaththri Vimalathas | 2025 | 111 | 31 | 80 |
| Supanut Kumjan | 2025 | 177 | 113 | 64 |
| Lili Wu | 2025 | 31 | 17 | 14 |
| Jing Yuan Tan | 2025 | 72 | 35 | 37 |
| Naree Warnnissorn | 2024 | 111 | 43 | 68 |
| Yohei Sasaki | 2024 | 128 | 28 | 86 |
| Jenny Coelho | 2024 | 112 | 32 | 80 |
| Tingting Yuan | 2024 | 289 | 35 | 167 |
| Phuttirak Yimpak | 2024 | 100 | 20 | 80 |
| Yi-Zhuo Chen | 2024 | 130 | 44 | 86 |
| Yanjie Wang | 2023 | 194 | 106 | 88 |
| Tan JY | 2023 | 72 | 28 | 44 |
| AlekseiK.Koviazin | 2023 | 52 | 21 | 31 |
| Sugeshnee Pather | 2022 | 110 | 28 | 92 |
| Rene-Olivier Casasnovas | 2022 | 79 | 27 | 52 |
| Jing Zhan | 2022 | 150 | 75 | 75 |
| MartaRodríguez | 2022 | 197 | 19 | 178 |
| Jin Roh | 2022 | 353 | 112 | 242 |
| Jin Roh | 2022 | 269 | 82 | 187 |
| Annalisa Chiappella | 2022 | 185 | 39 | 146 |
| Jin Roh | 2022 | 353 | 112 | 241 |
| Shoichi Kimura | 2022 | 111 | 37 | 74 |
| Dao‑guang Chen | 2021 | 56 | 34 | 22 |
| Xin Yang | 2021 | 163 | 42 | 121 |
| Denisse Castro | 2021 | 73 | 26 | 47 |
| Mu‑Chen Zhang | 2020 | 49 | 12 | 37 |
| Jie Xu | 2020 | 125 | 53 | 72 |
| Zhiping Ma | 2020 | 98 | 18 | 80 |
| Yu Ri Kim | 2020 | 67 | 23 | 44 |
| Bogyeong Han | 2020 | 388 | 108 | 280 |
| Maysaa Abdulla | 2020 | 249 | 47 | 108 |
| Yu Ri Kim | 2020 | 67 | 23 | 44 |
| Kean Chang PHANG | 2019 | 65 | 14 | 51 |
| Xin‑Yu Zhang | 2019 | 398 | 53 | 136 |
| Sixia Huang | 2019 | 130 | 51 | 79 |
| Allison Barraclough | 2019 | 175 | 37 | 138 |
| Linyu Li | 2018 | 212 | 42 | 170 |
| Alex F. Herrera | 2018 | 78 | 37 | 41 |
| Joshua Allen | 2018 | 167 | 26 | 141 |
| Ching Soon Teoh | 2018 | 71 | 35 | 36 |
| Katharina T Prochazka | 2018 | 117 | 60 | 57 |
| Wenjuan Yu | 2017 | 223 | 64 | 159 |
| Annette M. Staiger | 2017 | 57 | 25 | 32 |
| Wenli Yan | 2017 | 148 | 37 | 111 |
| Alex F. Herrera | 2017 | 117 | 47 | 58 |
| Ichiro Kawashima | 2017 | 60 | 37 | 23 |
| Hiromichi Takahashi | 2016 | 40 | 10 | 30 |
| Anne-Segolene Cottereau | 2016 | 57 | 9 | 48 |
| Katsuhiro Miura | 2015 | 38 | 17 | 21 |
| Kelli M. Clark Schneider | 2015 | 69 | 10 | 59 |
| Ting-Xun Lu | 2015 | 141 | 9 | 166 |
| David W. Scott | 2015 | 330 | 103 | 227 |
| Idun Fiskvik | 2015 | 67 | 6 | 43 |
| Anamarija M. Perry | 2014 | 62 | 47 | 15 |
| Carmen Bellas | 2014 | 100 | 21 | 79 |
| Eun Ji Oh | 2014 | 224 | 34 | 147 |
| Shimin Hu | 2013 | 466 | 157 | 309 |
| Nathalie A. Johnson, | 2012 | 167 | 28 | 136 |
|  |  | 140 | 36 | 104 |

Table S2B Sample size for DEL-TP53+ vs DEL-TP53-

| Another | Year | N | N(DEL-TP53+) | N(DEL-TP53-) |
| --- | --- | --- | --- | --- |
| XiaYinMS | 2025 | 33 | 12 | 21 |
| Yi-­ Fan Wu (1) | 2025 | 62 | 13 | 49 |
| Juan Carlos | 2023 | 38 | 21 | 17 |
| Anna Dodero | 2022 | 69 | 16 | 53 |
| Pu Huang | 2019 | 45 | 7 | 38 |

Table S2C Sample size for DEL-high IPI vs DEL-low IPI

| Another | Year | N | N(DEL-high IPI) | N(DEL-low IPI) |
| --- | --- | --- | --- | --- |
| XiaYinMS | 2025 | 48 | 20 | 28 |
| Yi-­ Fan Wu | 2025 | 111 | 49 | 62 |
| Xi Chen | 2024 | 62 | 29 | 33 |
| Sung H-J, | 2023 | 153 | 119 | 34 |
| Bogyeong Han | 2020 | 108 | 52 | 56 |
| Anna Dodero | 2022 | 122 | 67 | 55 |
| Bingjie Fan | 2021 | 152 | 60 | 92 |
| Pu Huang | 2019 | 45 | 17 | 28 |

Table S2D Sample size for DEL-DHL+ vs DEL-non DHL

| Another | Year | N | N(DEL-DHL) | N(DEL-non DHL) |
| --- | --- | --- | --- | --- |
| Anna Dodero | 2022 | 122 | 19 | 103 |
| Taha Al-Juhaishi | 2023 | 169 | 8 | 161 |

Table S2E Sample size for DEL-Age（>60）+ vs DEL- Age（<60）

| Another | Year | N | N(DEL-DHL) | N(DEL-non DHL) |
| --- | --- | --- | --- | --- |
| XiaYinMS | 2025 | 48 | 22 | 26 |
| Yi-­ Fan Wu (1) | 2025 | 62 | 38 | 24 |
| Yi-­ Fan Wu | 2025 | 277 | 175 | 102 |
| Xi Chen | 2024 | 62 | 23 | 39 |
| Sung H-J, | 2023 | 153 | >50% | <50% |
| Pu Huang | 2019 | 45 | 23 | 22 |
| Bingjie Fan | 2021 | 152 | 77 | 75 |
| Sirapat Rungwittayatiwat | 2021 | 87 | 63 | 24 |
| Bogyeong Han | 2020 | 108 | >50% | <50% |

Table S2F Sample size for DEL-Female+ vs DEL-Male

| Another | Year | N | N(DEL-DHL) | N(DEL-non DHL) |
| --- | --- | --- | --- | --- |
| XiaYinMS | 2025 | 48 | 19 | 29 |
| Xi Chen | 2024 | 62 | 32 | 30 |
| Anna Dodero | 2022 | 122 | 47 | 75 |
| Bingjie Fan | 2021 | 152 | 70 | 82 |
| Pu Huang | 2019 | 174 | 45 | 27 |
| Bogyeong Han | 2020 | 108 | 50 | 58 |

Table S2G Sample size for DEL-ECOG>2+ vs DEL- ECOG<2

| Another | Year | N | N(DEL-DHL) | N(DEL-non DHL) |
| --- | --- | --- | --- | --- |
| XiaYinMS | 2025 | 48 | - | - |
| Xi Chen | 2024 | 62 | 8 | 54 |
| Sirapat Rungwittayatiwat | 2021 | 87 | 5 | 82 |
| Bogyeong Han | 2020 | 87 | 17 | 70 |
| Bingjie Fan | 2021 | 152 | 47 | 105 |
| Yi-­ Fan Wu (1) | 2025 | 62 | 14 | 48 |

Table S2H Sample size for DEL-Ann(III/IV)+ vs DEL- Ann(I/II)

| Another | Year | N | N(DEL-DHL) | N(DEL-non DHL) |
| --- | --- | --- | --- | --- |
| Xi Chen | 2024 | 62 | 39 | 23 |
| Pu Huang | 2019 | 65 | 45 | 20 |
| Sirapat Rungwittayatiwat | 2021 | 87 | 48 | 39 |
| Anna Dodero | 2022 | 122 | 27 | 95 |
| Bogyeong Han | 2020 | 106 | 70 | 36 |
| Bingjie Fan | 2021 | 152 | 85 | 67 |
| Yi-­ Fan Wu (1) | 2025 | 62 | 42 | 20 |
| Yi-­ Fan Wu | 2025 | 275 | 190 | 85 |

Table S2I Sample size for DEL-With Extranodal involvement+ vs DEL-Without

| Another | Year | N | N(DEL-DHL) | N(DEL-non DHL) |
| --- | --- | --- | --- | --- |
| XiaYinMS | 2025 | 48 | 18 | 30 |
| Xi Chen | 2024 | 62 | 53 | 9 |
| Sirapat Rungwittayatiwat | 2021 | 87 | 54 | 33 |
| Bogyeong Han | 2020 | 108 | 39 | 69 |
| Yi-­ Fan Wu | 2025 | 281 | 72 | 209 |

Table S2J Sample size for DEL-LDH(rise)+ vs DEL- LDH(normal)

| Another | Year | N | N(DEL-DHL) | N(DEL-non DHL) |
| --- | --- | --- | --- | --- |
| XiaYinMS | 2025 | 48 | 28 | 20 |
| Xi Chen | 2024 | 62 | 27 | 35 |
| Pu Huang | 2019 | 174 | 45 | 22 |
| Sirapat Rungwittayatiwat | 2021 | 87 | 56 | 31 |
| Bogyeong Han | 2020 | 102 | 67 | 35 |
| Bingjie Fan | 2021 | 152 | 53 | 99 |
| Yi-­ Fan Wu (1) | 2025 | 62 | 37 | 25 |
| Yi-­ Fan Wu | 2025 | 248 | 131 | 117 |

Table S2K Sample size for DEL-with B symptom+ vs DEL- without B symptom

| Another | Year | N | N(DEL-DHL) | N(DEL-non DHL) |
| --- | --- | --- | --- | --- |
| Xi Chen | 2024 | 62 | 21 | 41 |
| Sung H-J, | 2023 | 153 | 26 | 127 |
| Bogyeong Han | 2020 | 108 | 11 | 97 |

Table S2L Sample size for DEL-GCB+ vs DEL-non GCB

| Another | Year | N | N(DEL-DHL) | N(DEL-non DHL) |
| --- | --- | --- | --- | --- |
| XiaYinMS | 2025 | 48 | 28 | 20 |
| Xi Chen | 2024 | 62 | 27 | 35 |
| Pu Huang | 2019 | 174 | 45 | 22 |
| Sirapat Rungwittayatiwat | 2021 | 87 | 56 | 31 |
| Bogyeong Han | 2020 | 102 | 67 | 35 |
| Bingjie Fan | 2021 | 152 | 124 | 28 |
| Yi-­ Fan Wu | 2025 | 226 | 123 | 103 |

Table S2M Sample size for DEL-Ki-67(High) vs DEL--Ki-67(Low)

| Another | Year | N | N(DEL-DHL) | N(DEL-non DHL) |
| --- | --- | --- | --- | --- |
| Xi Chen | 2024 | 60 | 49 | 11 |
| Bingjie Fan | 2021 | 142 | 112 | 40 |

Figure S1 Funnel Plot of PFS and OS

Figure S1A Funnel Plot of PFS for DEL vs non-DEL

Figure S1B Funnel Plot of OS for DEL vs non-DEL

Figure S1C Funnel Plot of PFS for DEL-TP53(+) vs DEL-TP53(-)

Figure S1D Funnel Plot of OS for DEL-TP53(+) vs DEL-TP53(-)

Figure S1E Funnel Plot of PFS for DEL-high IPI vs DEL-low IPI

Figure S1F Funnel Plot of OS for DEL-high IPI vs DEL-low IPI

Figure S1G Funnel Plot of PFS for DEL-DHL(+) VS DEL-DHL(-)

Figure S1H Funnel Plot of OS for DEL-DHL(+) VS DEL-DHL(-)

Figure S1I Funnel Plot of PFS for DEL-Age（>60）VS Age（<60）

Figure S1J Funnel Plot of OS for DEL-Age（>60）VS Age（<60）

Figure S1K Funnel Plot of PFS for DEL- Female VS Male

Figure S1L Funnel Plot of OS for DEL- Female VS Male

Figure S1M Funnel Plot of PFS for DEL- ECOG>2 VS ECOG 1/2

Figure S1N Funnel Plot of OS for DEL- ECOG>2 VS ECOG 1/2

Figure S1O Funnel Plot of PFS for DEL-Ann（III/IV）VS Ann（I/II）

Figure S1P Funnel Plot of OS for DEL-Ann（III/IV）VS Ann（I/II）

Figure S1Q Funnel Plot of PFS for DEL- With Extranodal involvement VS Without

Figure S1R Funnel Plot of OS for DEL- With Extranodal involvement VS Without

Figure S1S Funnel Plot of PFS for DEL- LDH（rise）VS LDH（normal）

Figure S1T Funnel Plot of OS for DEL- LDH（rise）VS LDH（normal）

Figure S1U Funnel Plot of PFS for DEL- With B Symptom VS Without B Symptom

Figure S1V Funnel Plot of OS for DEL- With B Symptom VS Without B Symptom

Figure S1W Funnel Plot of PFS for DEL- GCB VS non- GCB

Figure S1X Funnel Plot of OS for DEL- GCB VS non- GCB

Figure S1Y Funnel Plot of PFS for DEL-Ki-67(High) vs DEL--Ki-67(Low)

Figure S1Z Funnel Plot of OS for DEL-Ki-67(High) vs DEL--Ki-67(Low)

Table S3 Sample size for comparison of treatment

| Another Year | Experimental group | | | Control group | | |
| --- | --- | --- | --- | --- | --- | --- |
|  | Treatment | N(CR) | N(all) | Treatment | N(CR) | N(all) |
| Min Zhang 2025 | ZR-CHOP | 19 | 23 | R-CHOP | 30 | 55 |
| Demei Feng 2025 | R2-CHOP | 52 | 65 | R-CHOP | 78 | 112 |
|  | R-CHOP+BKI | 27 | 36 | R-CHOP | 78 | 112 |
| Jeremy S. Abramson2024 | R-CHOP + Ven | 47 | 57 | R-CHOP | 39 | 56 |
| Ting Deng2024 | R-CHOP+BTKI | 9 | 10 | R-CHOP | 4 | 9 |
| Weili Zhao2024 | CR-CHOP | 154 | 211 | R-CHOP | 131 | 212 |
| Peter W. M. Johnson2023 | I+R-CHOP | 83 | 123 | R-CHOP | 72 | 111 |
| JunZhu2022 | I+R-CHOP | 27 | 42 | R-CHOP | 23 | 38 |
| Jing Zhan2022 | DA-EPOCH-R | 17 | 26 | R-CHOP | 25 | 49 |
| Shuhan Tang2022 | REPOCH | 42 | 65 | R-CHOP | 35 | 82 |
| Tamer Othman2022 | DA-EPOCH-R | 69 | 94 | R-CHOP | 45 | 61 |
| C. R. D’Angelo2021 | DA-EPOCH-R | 31 | 44 | R-CHOP | 34 | 46 |
| A. Dodero2019 | DA-EPOCH-R | 37 | 51 | R-CHOP | 44 | 63 |
| Mette ØlgodPedersen2017 | DA-EPOCH-R | 24 | 26 | R-CHOP | 30 | 36 |

|  | Min Zhang 2025  ZR-CHOP vs R-CHOP | | | | Demei Feng 2025  R2-CHOP/R-CHOP+BKI vs R-CHOP | | | | | | Ting Deng 2024  R-CHOP+BKI vs R-CHOP | | | |
| --- | --- | --- | --- | --- | --- | --- | --- | --- | --- | --- | --- | --- | --- | --- |
|  | Experimental | | Control | | Exp 1 | | Exp 2 | | Control | | Experimental | | Control | |
|  | N1 | N(all) | N1 | N(all) | N1 | N(all) | N1 | N(all) | N1 | N(all) | N1 | N(all) | N1 | N(all) |
| Hematologic (Grade3-4) | | | | | | | | | | | | | | |
| Anemia | 11 | 23 | 26 | 55 | 2 | 65 | 4 | 36 | 14 | 112 | 9 | 18 | 8 | 17 |
| Thrombocytopenia | 7 | 23 | 28 | 55 | 12 | 65 | 6 | 36 | 11 | 112 | 7 | 18 | 7 | 17 |
| Neutropenia | 9 | 23 | 30 | 55 | 33 | 65 | 11 | 36 | 48 | 112 | 8 | 18 | 7 | 17 |
| Febrile neutropenia | 6 | 23 | 13 | 55 | 0 | 65 | 0 | 36 | 0 | 112 | 3 | 18 | 4 | 17 |
| Leukopenia |  |  |  |  | 33 | 65 | 15 | 36 | 57 | 112 |  |  |  |  |
| Non-hematological adverse events | | | | | | | | | | | | | | |
| Infection | 9 | 23 | 17 | 55 | 1 | 65 | 0 | 36 | 12 | 112 | 4 | 18 | 6 | 17 |
| Atrial fibrillation | 0 | 23 | 1 | 55 |  |  |  |  |  |  | 3 | 18 | 2 | 17 |
| Hemorrhage/Ecchymosis | 1 | 23 | 4 | 55 |  |  |  |  |  |  | 0 | 18 | 0 | 17 |
| Hyperuricemia | 4 | 23 | 19 | 55 |  |  |  |  |  |  |  |  |  |  |
| Elevated transaminases | 6 | 23 | 21 | 55 | 26 | 65 | 14 | 36 | 37 | 112 | 7 | 18 | 10 | 17 |
| Nausea and vomiting | 6 | 23 | 23 | 55 | 1 | 65 | 0 | 36 | 2 | 112 |  |  |  |  |
| Anorexia | 6 | 23 | 17 | 55 |  |  |  |  |  |  |  |  |  |  |
| Diarrhea | 2 | 23 | 9 | 55 | 2 | 65 | 0 | 36 | 2 | 112 | 5 | 18 | 2 | 17 |
| Fatigue | 8 | 23 | 32 | 55 |  |  |  |  |  |  |  |  |  |  |
| Elevated bilirubin |  |  |  |  | 5 | 65 | 5 | 36 | 5 | 112 |  |  |  |  |
| Decreased albumin |  |  |  |  | 13 | 65 | 11 | 36 | 34 | 112 |  |  |  |  |
| Rash |  |  |  |  | 8 | 65 | 4 | 36 | 4 | 112 |  |  |  |  |
| Sensory neuropathy |  |  |  |  | 9 | 65 | 3 | 36 | 13 | 112 |  |  |  |  |
| Hematologic (Grade3-4) |  |  |  |  | 3 | 65 | 1 | 36 | 4 | 112 |  |  |  |  |
| Anemia |  |  |  |  |  |  |  |  |  |  | 1 | 18 | 2 | 17 |
| Table S4 Adverse Reactions Associated with Various Treatment Regimens | | | | | | | | | | | | | | |

|  | Jun Zhu 2022  R-CHOP+I vs R-CHOP | | | | Jing Zhan 2022  DA-EPOCH-R vs RCHOP | | | | Jeremy S. Abramson 2024  Ven- RCHOP vs RCHOP | | | |
| --- | --- | --- | --- | --- | --- | --- | --- | --- | --- | --- | --- | --- |
|  | Experimental | | Control | | Experimental | | Control | | Experimental | | Control | |
|  | N1 | N(all) | N1 | N(all) | N1 | N(all) | N1 | N(all) | N1 | N(all) | N1 | N(all) |
| Hematologic (Grade3-4) | | | | | | | | | | | | |
| Anemia |  |  |  |  | 9 | 43 | 12 | 107 | 15 | 57 | 2 | 56 |
| Thrombocytopenia | 62 | 103 | 64 | 96 | 13 | 43 | 15 | 107 | 14 | 57 | 3 | 56 |
| Neutropenia | 22 | 103 | 13 | 96 | 28 | 43 | 40 | 107 | 27 | 57 | 20 | 56 |
| Febrile neutropenia | 36 | 103 | 35 | 96 | 12 | 43 | 22 | 107 | 9 | 57 | 4 | 56 |
| Leukopenia |  |  |  |  |  |  |  |  |  |  |  |  |
| Non-hematological adverse events | | | | | | | | | | | | |
| Infection |  |  |  |  |  |  |  |  |  |  |  |  |
| Atrial fibrillation | 4 | 103 | 1 | 96 |  |  |  |  |  |  |  |  |
| Hemorrhage/Ecchymosis |  |  |  |  |  |  |  |  |  |  |  |  |
| Hyperuricemia |  |  |  |  |  |  |  |  |  |  |  |  |
| Elevated transaminases |  |  |  |  |  |  |  |  |  |  |  |  |
| Nausea and vomiting |  |  |  |  |  |  |  |  |  |  |  |  |
| Anorexia |  |  |  |  |  |  |  |  |  |  |  |  |
| Diarrhea |  |  |  |  |  |  |  |  |  |  |  |  |
| Fatigue |  |  |  |  |  |  |  |  | 6 | 57 | 0 | 56 |
| Elevated bilirubin |  |  |  |  |  |  |  |  |  |  |  |  |
| Decreased albumin |  |  |  |  |  |  |  |  |  |  |  |  |
| Rash |  |  |  |  |  |  |  |  |  |  |  |  |
| Sensory neuropathy |  |  |  |  |  |  |  |  |  |  |  |  |
| Hematologic (Grade3-4) |  |  |  |  |  |  |  |  |  |  |  |  |
| Anemia |  |  |  |  |  |  |  |  |  |  |  |  |
| Table S4 Adverse Reactions Associated with Various Treatment Regimens | | | | | | | | | | | | |

Figure S7 Forest Plot of Adverse Reactions for the R-CHOP+BKI Regimen


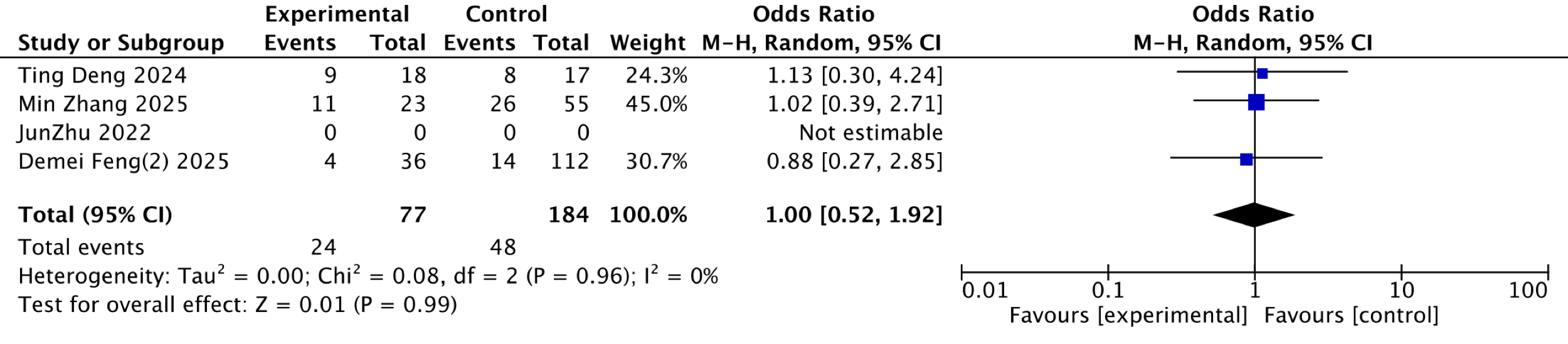


Figure S7A Anemia


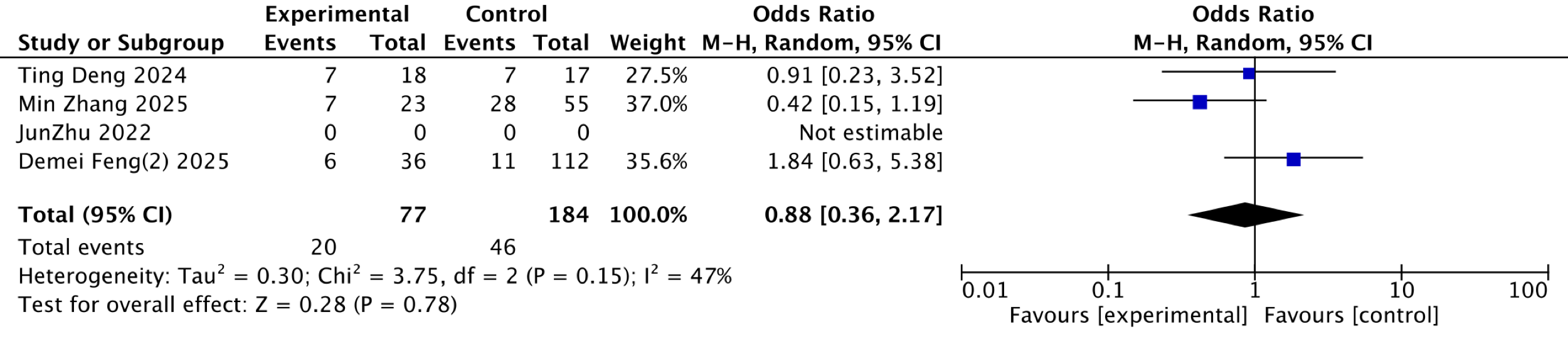


Figure S7B Thrombocytopenia


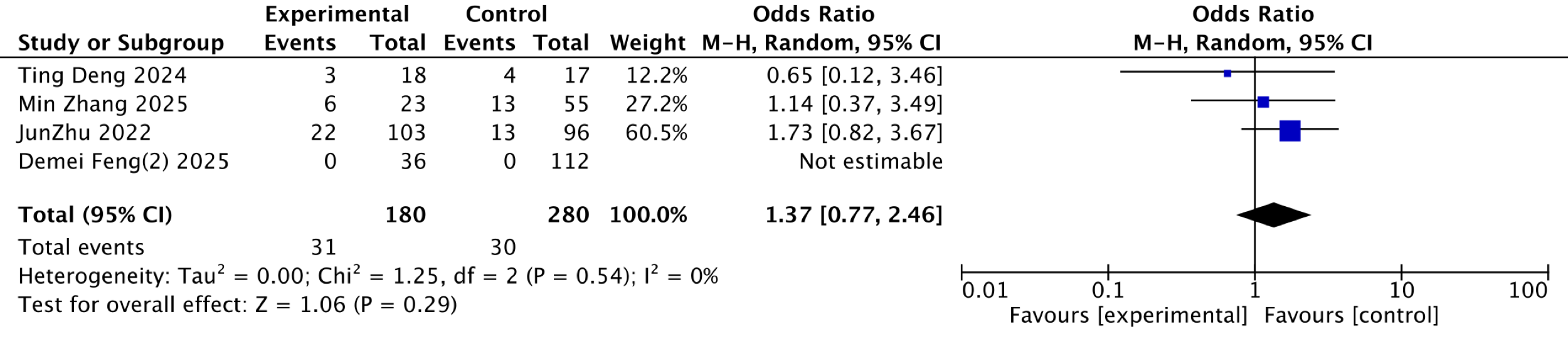


Figure S7C Neutropenia


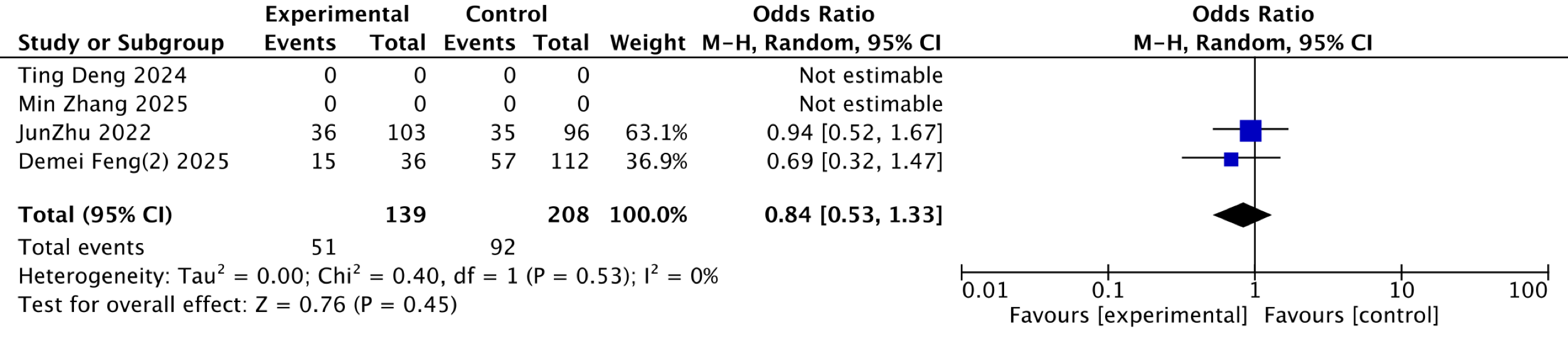


Figure S7D Leukopenia


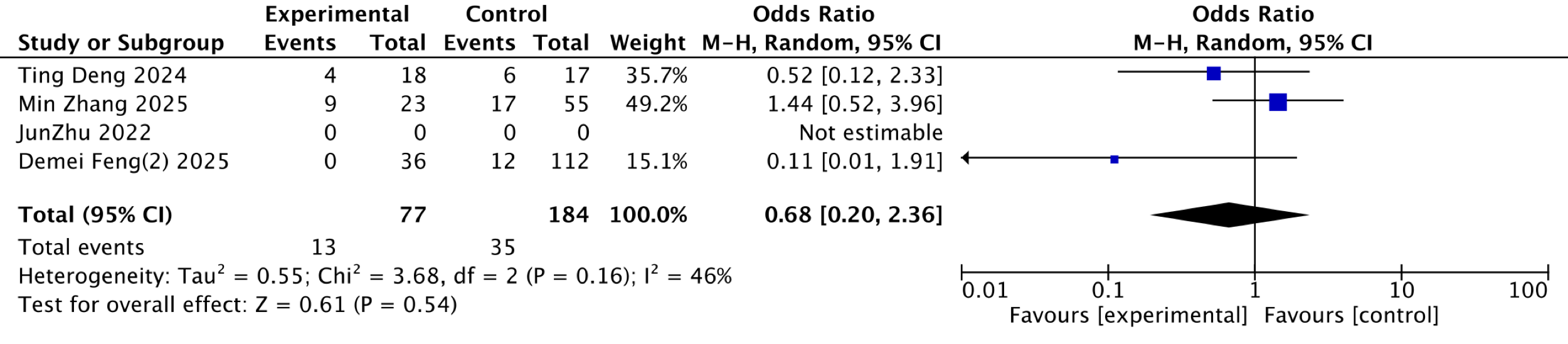


Figure S7E Leukopenia


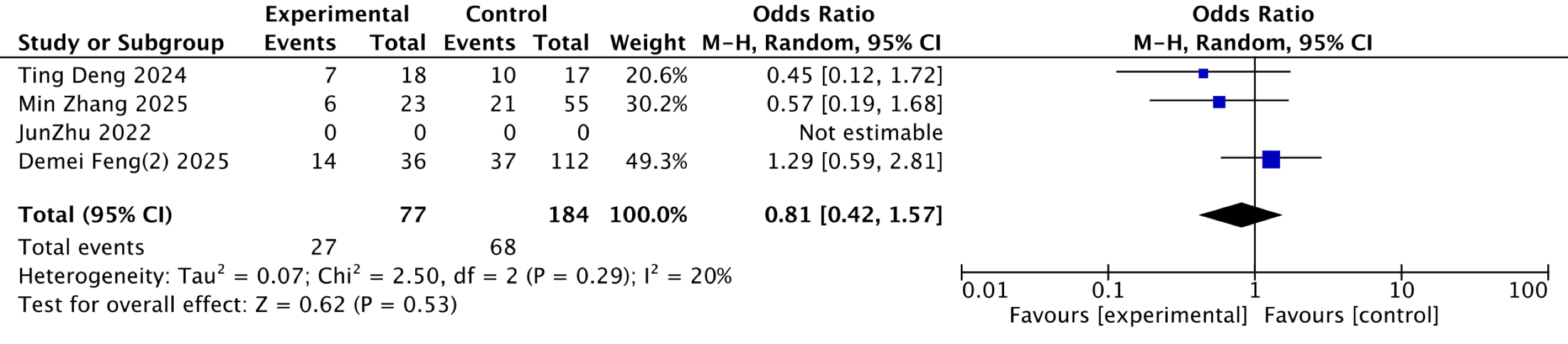


Figure S7F Elevated transaminases


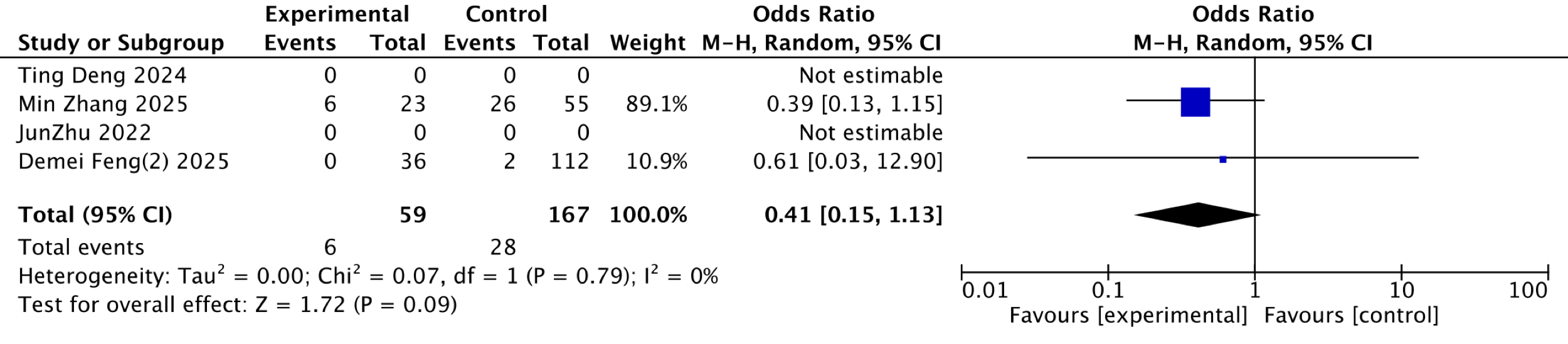


Figure S7G Nausea and vomiting


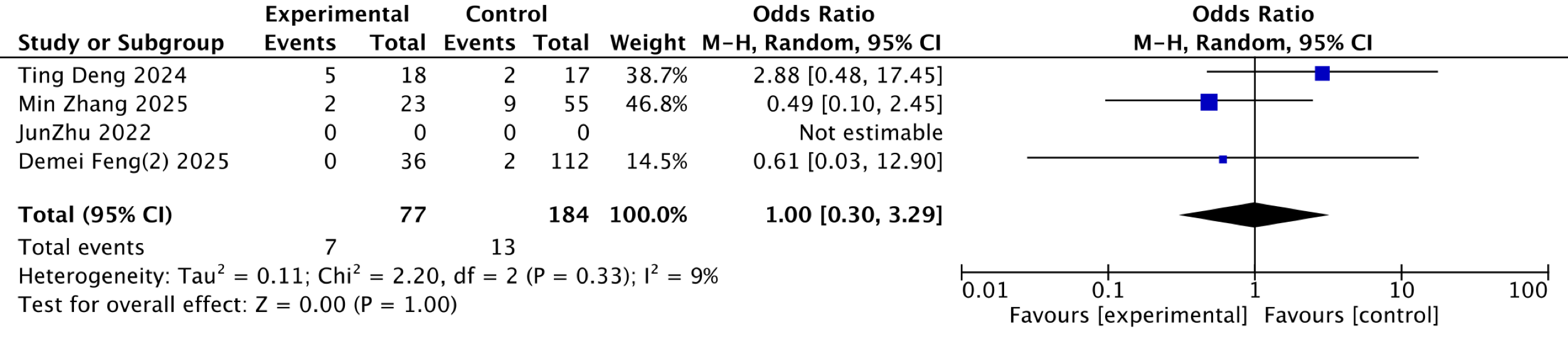


Figure S7H Diarrhea
